# Structural insights into the divergent evolution of a photosystem I supercomplex in *Euglena gracilis*

**DOI:** 10.1126/sciadv.aea6241

**Published:** 2025-10-31

**Authors:** Koji Kato, Yoshiki Nakajima, Runa Sakamoto, Minoru Kumazawa, Kentaro Ifuku, Takahiro Ishikawa, Jian-Ren Shen, Atsushi Takabayashi, Ryo Nagao

**Affiliations:** ^1^ Research Institute for Interdisciplinary Science, Advanced Research Field, and Graduate School of Environmental, Life, Natural Science and Technology, Okayama University, Okayama, Okayama 700-8530, Japan.; ^2^ Institute of Low Temperature Science, Hokkaido University, Sapporo, Hokkaido 060-0819, Japan.; ^3^ Graduate School of Agriculture, Kyoto University, Kyoto, Kyoto 606-8502, Japan.; ^4^ Institute of Agricultural and Life Sciences, Academic Assembly, Shimane University, Matsue, Shimane 690-8504, Japan.; ^5^ Faculty of Agriculture, Shizuoka University, Shizuoka, Shizuoka 422-8529, Japan.

## Abstract

Photosystem I (PSI) forms supercomplexes with light-harvesting complexes (LHCs) to perform oxygenic photosynthesis. Here, we report a 2.82-angstrom cryo–electron microscopy structure of the PSI-LHCI supercomplex from *Euglena gracilis*, a eukaryotic alga with secondary green alga-derived plastids. The structure reveals a PSI monomer core with eight subunits and 13 asymmetrically arranged LHCI proteins. *Euglena* LHCIs bind diadinoxanthin, which is one of the carotenoids typically associated with red-lineage LHCs and is not present in the canonical LHCI belt found in green-lineage PSI-LHCI structures. Phylogenetic analysis shows that the *Euglena* LHCIs originated from LHCII-related clades rather than from the green-lineage LHCI group and that the nuclear-encoded PSI subunit PsaD likely originated from cyanobacteria via horizontal gene transfer. These observations indicate a mosaic origin of the *Euglena* PSI-LHCI. Our findings uncover a noncanonical light-harvesting architecture and highlight the structural and evolutionary plasticity of photosynthetic systems, illustrating how endosymbiotic acquisition and lineage-specific adaptation shape divergent light-harvesting strategies.

## Teaser

The structure of *Euglena* PSI-LHCI reveals a unique, chimeric light-harvesting system distinct from green and red lineages.

## INTRODUCTION

Oxygenic photosynthesis conducted by cyanobacteria, algae, and land plants converts light energy into chemical energy while releasing molecular oxygen as a by-product (*1*). This fundamental biological process is executed by two highly organized membrane-embedded protein assemblies—photosystem I (PSI) and photosystem II (PSII)—which function independently to initiate distinct charge-separation events yet operate cooperatively to sustain sequential electron transport (*2*–*5*). Various light-harvesting antennas bind to the PSI and PSII cores and optimize the capture of solar energy and transfer of excitation energy to the photosystems in oxyphototrophs (*1*). Photosynthetic antenna systems display remarkable structural and compositional diversity across taxa and are broadly categorized into integral membrane complexes and soluble proteins (*1*). The former group is dominated by the light-harvesting complex (LHC) protein superfamily (*6*, *7*), which uses various chlorophylls (Chls) and carotenoids (Cars) to absorb light.

Species-specific variation in pigments and stoichiometries among LHCs contributes to the spectral and visual heterogeneity observed across photosynthetic lineages, which are evolutionarily divided into the green and red lineages (*8*). The green lineage comprises green algae, land plants, and some secondary endosymbiotic algae, whereas the red lineage encompasses red algae and secondary endosymbiotic algae harboring chloroplasts of red algal origin, including cryptophytes, diatoms, haptophytes, and dinoflagellates (*8*). In eukaryotes, PSI associates with a distinct set of LHC subunits (LHCIs) to form the PSI-LHCI supercomplex, whose three-dimensional (3D) architecture has been resolved in multiple species by cryo–electron microscopy (cryo-EM) (*9*–*13*).

*Euglena* is a genus of secondary endosymbiotic eukaryotes that acquired chloroplasts through secondary endosymbiosis with a green alga. Phylogenetic analysis of chloroplast genomes indicates that the chloroplasts of Euglenophyceae originated from an ancestral green alga belonging to the order Pyramimonadales in the class Prasinophyceae (*14*, *15*), an early diverging lineage of Chlorophyta commonly referred to as green algae. In addition, the nuclear genome of *Euglena* is remarkably complex, shaped not only by endosymbiotic gene transfer (EGT) from the endosymbiont genome to the host nuclear genome but also by multiple horizontal gene transfers (HGTs) from diverse organisms, including bacteria and other algal lineages. Proposed mechanisms for such HGT include pathways via transient endosymbiosis as well as direct gene transfer independent of endosymbiosis (*16*). As a result, the nuclear genome of *Euglena* contains numerous genes derived not only from its green algal endosymbiont but also from red-lineage algae and various other organisms, giving rise to a mosaic genome structure (*16*–*19*).

*Euglena gracilis*, a representative model organism of *Euglena*, grows photoautotrophically, and its PSI-related genes are encoded separately in the chloroplast and nuclear genomes. Although the chloroplast genome of *E. gracilis* has already been sequenced, the nuclear genome remains only partially resolved owing to its large size and complex transcriptional organization. The chloroplast genome encodes five PSI subunits: PsaA, PsaB, PsaC, PsaJ, and PsaM (*14*). Because the *E. gracilis* chloroplast genome is highly similar to that of *Pyramimonas parkeae*, an extant green alga in the order Pyramimonadales thought to be closely related to its ancestral lineage (*15*), these PSI subunits are presumed to be derived from the endosymbiotic green alga. By contrast, three nuclear-encoded PSI subunits—PsaD, PsaE, and PsaF—have been predicted to be present in *E. gracilis* (*20*). This number of PSI subunits is notably smaller than that found in most green algae, such as *Chlamydomonas reinhardtii* (*20*), suggesting substantial structural remodeling of PSI associated with secondary endosymbiosis. Phylogenetic analysis of these three nuclear-encoded subunits has rarely been conducted. However, PsaE and PsaF of *E. gracilis* exhibit high sequence similarity to their counterparts in the green alga *Chlamydomonas* (*21*), supporting a green algal origin.

In contrast to other green algae and land plants, LHCs in *Euglena* have followed a distinct evolutionary trajectory. With the exception of CP29 (LHCB4), *Euglena* LHCs are thought to have diverged from a limited subset of LHCIIs (LHCBs) typically associated with PSII (*22*). As LHCs are encoded in the nuclear genome, the basal LHC genes were likely acquired from green algae via EGT or HGT early in the plastid acquisition. Molecular evidence for the unique evolution of *Euglena* LHCs includes tandem gene duplications that produced polycistronic transcriptional and translational units encoding multiple LHCs, which are thought to have expanded further through repeated duplication events (*23*). Notably, *Euglena* lacks the LHCI gene group (LHCAs) (*22*), leading to the hypothesis that certain LHCs derived from the LHCII group may functionally replace LHCI by associating with PSI. Unlike in green algae and land plants, PSI-binding LHCs cannot be inferred in *Euglena* from sequence or phylogenetic analyses, suggesting a fundamentally reorganized light-harvesting system. This reorganization may reflect “neolocalization,” a phenomenon in which LHC subunits are evolutionarily rearranged through genome reduction and endosymbiosis, recently recognized as diverse and flexible (*24*–*26*). Thus, the evolutionary origins of PSI subunits encoded in the *Euglena* nuclear genome, the identity of PSI-associated LHCs, and their molecular diversification remain poorly understood.

In this study, we determined the cryo-EM structure of PSI-LHCI from *E. gracilis* strain Z at a 2.82-Å resolution using single-particle analysis. The structure reveals a PSI-monomer core and 13 LHCI subunits. Phylogenetic analysis based on this structure indicates that these subunits occupy an evolutionarily singular position, underscoring the unique mosaic origin of *Euglena* photosynthesis and its marked divergence from primary photosynthetic lineages.

## RESULTS

### Structure of the PSI-LHCI supercomplex from *E. gracilis* strain Z

**Fig. 1.**
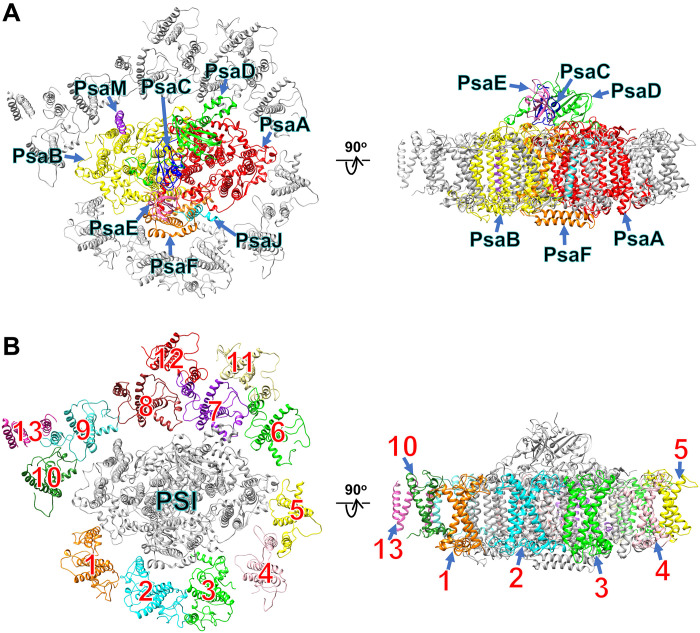
Overall structure of the PSI-LHCI supercomplex from *E. gracilis*. Structures are viewed from the stromal side (left panels) and the direction perpendicular to the membrane normal (right panels). Only protein structures are shown, and cofactors are omitted for clarity. The PSI-core (**A**) and LHCI (**B**) subunits are labeled and colored differently. The numbers 1 to 13 correspond to LHCI-1 to LHCI-13, respectively, in (B).

Cryo-EM analysis of the *Euglena* PSI-LHCI supercomplex yielded a 2.82-Å map with C1 symmetry (figs. S1 and S2 and table S1). The atomic model of PSI-LHCI was built (see Materials and Methods, fig. S2, and tables S1 to S3), and the structure reveals a monomeric PSI core composed of eight subunits, surrounded by 13 LHCI subunits (Fig. 1, A and B). The eight core subunits are PsaA, PsaB, PsaC, PsaD, PsaE, PsaF, PsaJ, and PsaM in the *E. gracilis* PSI-LHCI (Fig. 1A). Compared with the structures of PSI supercomplexes from land plants and green algae (*9*–*13*), the *E. gracilis* PSI lacks PsaG, PsaH, PsaI, PsaK, PsaL, PsaN, and PsaO. The PSI core of *E. gracilis* includes 89 Chls *a*, 17 β-carotenes (BCRs), 3 [4Fe-4S] clusters, 2 phylloquinone molecules, and 4 lipid molecules (table S3).

The 13 LHCI subunits are designated LHCI-1 to LHCI-13 (Fig. 1B), following the PSI-LHCI structure of *Pisum sativum* (*27*). In particular, the name and binding site of LHCI-1 in *E. gracilis* correspond to those of LHCA1 in *P. sativum*. These subunits are asymmetrically distributed, with LHCI-1 to LHCI-5 positioned on the PsaF side and LHCI-6 to LHCI-13 on the opposite side (Fig. 1, A and B). Given that the comprehensive genome sequence of *E. gracilis* is not yet available, the subunits were modeled from transcriptome data (*16*, *17*, *28*) using ModelAngelo (see Materials and Methods for details) (*29*). As a result, gene names corresponding to these LHCIs remain undetermined. Subunit identity was assigned on the basis of characteristic amino acid residues discernible in the cryo-EM map (fig. S3). The root mean square deviations between the structure of LHCI-4 and those of the other 12 LHCIs range from 0.71 to 1.49 Å (table S4). LHCI-12 and LHCI-13 share the same sequence as LHCI-11 within the structurally resolved regions. However, owing to poor map quality, only partial models could be built, and their sequences are therefore omitted from fig. S3A.

These LHCIs include 148 Chls *a*, 26 diadinoxanthins (Ddxs), and 11 lipid molecules (table S3). High-performance liquid chromatography analysis confirmed the presence of Chl *b* in the PSI-LHCI supercomplex of *E. gracilis* (*30*), but no Chl *b* molecules could be assigned in the structure because of limited resolution in the LHCI region. The pigment composition for each LHCI subunit is summarized in fig. S4 and table S3. The axial ligands for the central Mg atoms of Chls are provided by amino acid residues, water molecules, and lipids, as listed in table S5.

### Structural comparisons of the binding properties of LHCIs

**Fig. 2.**
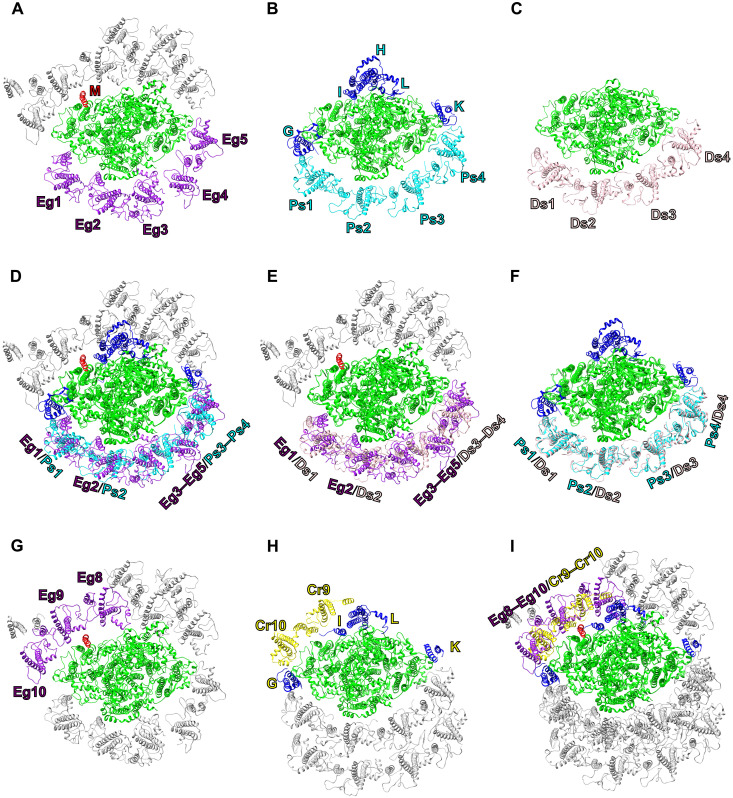
Structural comparisons of the *Euglena* PSI-LHCI with those of three green-lineage organisms. PSI-LHCI structures of *E. gracilis* (**A** and **G**), *P. sativum* (PDB: 7DKZ) (**B**), *D. salina* (PDB: 6RHZ) (**C**), and *C. reinhardtii* (PDB: 6JO5) (**H**) are viewed from the stromal side. Structures of PSI-LHCI are superimposed between *E. gracilis* and *P. sativum* (**D**), between *E. gracilis* and *D. salina* (**E**), between *P. sativum* and *D. salina* (**F**), and between *E. gracilis* and *C. reinhardtii* (**I**). Only protein structures are shown, and cofactors are omitted for clarity. The characteristic PSI-LHCI subunits are shown in different colors (excluding gray), with their corresponding labels indicated in the figure: green, PSI-core subunits conserved among the four species; red, PsaM (M); purple, LHCIs of *E. gracilis* (Eg1 to Eg5 and Eg8 to Eg10); blue, PsaG (G)/PsaH (H)/PsaI (I)/PsaK (K)/PsaL (L); cyan, LHCIs of *P. sativum* (Ps1 to Ps4); pink, LHCIs of *D. salina* (Ds1 to Ds4); yellow, LHCIs of *C. reinhardtii* (Cr9 and Cr10).

The LHCI-binding arrangement in the *Euglena* PSI-LHCI markedly differs from those previously elucidated in land plants and green algae (*9*–*13*). It is known that the PSI-LHCI structures of land plants and green algae have a well-ordered inner belt of four LHCIs at the side of PsaF and PsaJ in the PSI core (*9*–*13*). For the structural comparison of PSI-LHCI (Fig. 2), here, we selected the PSI-LHCI structure of *P. sativum* showing four LHCI subunits at the inner belt reported by x-ray crystallography (*31*, *32*), and that of the green alga *Dunaliella salina* showing a minimal PSI core complex containing only PsaA, PsaB, PsaC, PsaD, PsaE, PsaF, and PsaJ with four LHCIs (*33*).

The *E. gracilis* PSI-LHCI harbors five LHCIs at the positions of Eg1 to Eg5 corresponding to the inner belt (Fig. 2A), with binding patterns distinct from four LHCIs at the positions of Ps1 to Ps4 in the *P. sativum* PSI-LHCI (Fig. 2B) and of Ds1 to Ds4 in the *D. salina* PSI-LHCI (Fig. 2C). Two LHCIs at the Eg1 and Eg2 sites in the *E. gracilis* PSI-LHCI display a minor clockwise shift as seen from the stromal side compared with two LHCIs at the Ps1 and Ps2 sites in the *P. sativum* PSI-LHCI (Fig. 2D) and at the Ds1 and Ds2 sites in the *D. salina* PSI-LHCI (Fig. 2E). In addition, three LHCIs at the Eg3 to Eg5 sites in the *E. gracilis* PSI-LHCI are reoriented and compactly accommodated at the corresponding sites of Ps3 and Ps4 in the *P. sativum* PSI-LHCI (Fig. 2D) and of Ds3 and Ds4 in the *D. salina* PSI-LHCI (Fig. 2E). The binding properties of four LHCIs at the Ps1 to Ps4 sites in the *P. sativum* PSI-LHCI are very similar to those at the Ds1 to Ds4 sites in the *D. salina* PSI-LHCI (Fig. 2F). Thus, the *E. gracilis* PSI-LHCI does not have the inner belt of LHCIs formed at the side of PsaF and PsaJ in most green-lineage organisms.

The *P. sativum* PSI-LHCI shows PsaG, PsaH, PsaI, PsaK, and PsaL as PSI-core subunits (Fig. 2B), which are absent in the *E. gracilis* and *D. salina* PSI-LHCI structures (Fig. 2A, C). Among them, PsaG and PsaK are closely located near two LHCIs at the Ps1 and Ps4 sites, respectively (Fig. 2B). Despite the absence of PsaG and PsaK in the *D. salina* PSI-LHCI, the four LHCIs exhibit an arrangement highly similar to those in the *P. sativum* PSI-LHCI (Fig. 2, C and F). These observations suggest that the association of four LHCIs with PSI at the inner belt is not primarily dependent on PsaG and PsaK. In contrast, the *E. gracilis* PSI-LHCI exhibits unique interactions between LHCI and PSI, the details of which are described in the following section. Thus, the irregular and dispersed arrangement of *Euglena* LHCIs, rather than a well-ordered assembly along the inner belt of LHCIs in other green lineages, may be a characteristic feature of the diverse LHCI types found in red-lineage organisms, although *Euglena* belongs to the green lineage.

The other eight LHCI subunits are bound to the opposite side of PsaF and PsaJ in the *E. gracilis* PSI-LHCI (Fig. 1). Among them, three LHCIs at the sites of Eg8 to Eg10 are reoriented and compactly accommodated at the corresponding sites of Cr9 and Cr10 of the *C. reinhardtii* PSI-LHCI (Fig. 2, G to I). This feature is in line with the red-lineage PSI-LHCI supercomplexes, reinforcing the structural characteristics of *Euglena* LHCIs. The inner belt of LHCIs in the *C. reinhardtii* PSI-LHCI is virtually identical to that in the *P. sativum* PSI-LHCI.

### Interactions of LHCIs with PSI in *E. gracilis*

**Fig. 3.**
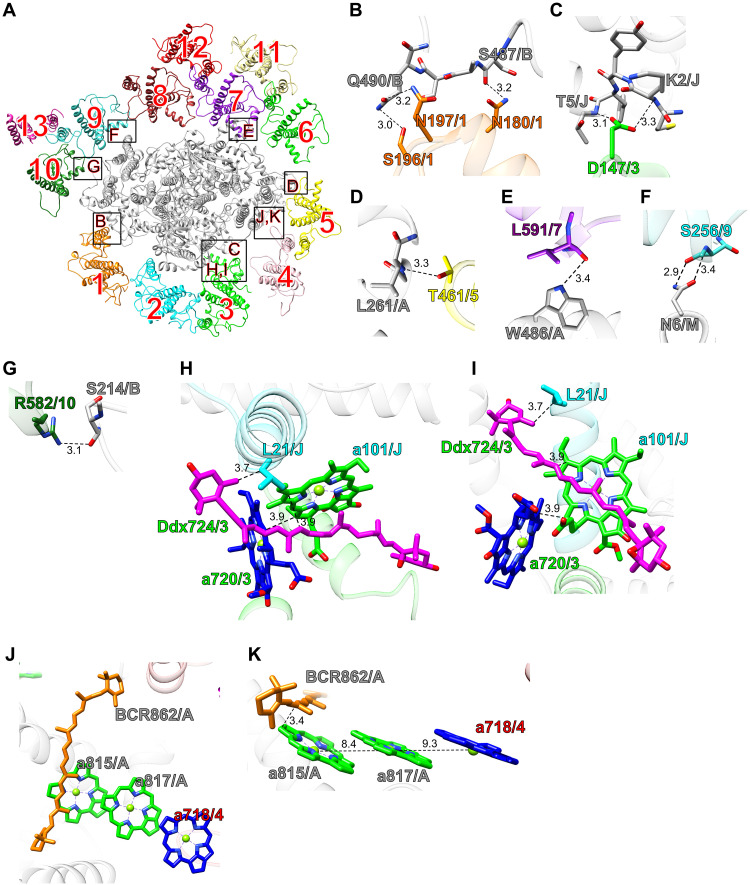
Characteristic structures in the *Euglena* PSI-LHCI. (**A**) The PSI-LHCI structure is viewed from the stromal side. The areas encircled by black squares are enlarged in (B) to (K). The numbers 1 to 13 correspond to LHCI-1 to LHCI-13, respectively. (**B** to **G**) Protein-protein interactions between PSI and LHCI subunits. Interactions are indicated by dashed lines, and the numbers are distances in Å. Amino acid residues participating in the interactions are labeled; for example, Q490/B and N180/1 indicate Gln^490^ of PsaB and Asn^180^ of LHCI-1, respectively. A, PsaA; B, PsaB; J, PsaJ; M, PsaM; 1, LHCI-1; 3, LHCI-3; 5, LHCI-5; 7, LHCI-7; 9, LHCI-9; 10, LHCI-10. (**H** and **I**) Pigment-pigment and pigment-protein interactions in the interface between PsaJ and LHCI-3 viewed from the stromal side (H) and from the direction perpendicular to the membrane normal (I). L21/J, Leu^21^ of PsaJ; a101/J, Chl a101 of PsaJ; a720/3, Chl a720 of LHCI-3; Ddx724/3, Ddx724 of LHCI-3. (**J** and **K**) Characteristic pigment molecules in the interface between PsaA and LHCI-4 viewed from the stromal side (J) and from the direction perpendicular to the membrane normal (K). BCR862/A, BCR862 of PsaA; a815/A, Chl a815 of PsaA; a817/A, Chl a817 of PsaA; a718/4, Chl a718 of LHCI-4.

Among LHCIs, LHCI-1, LHCI-3, LHCI-5, LHCI-7, LHCI-9, and LHCI-10 are likely involved in protein-protein interactions with PSI (Fig. 3, A to G), especially between N180/S196/N197 of LHCI-1 and S487/Q490 of PsaB at distances of 3.0 to 3.2 Å (Fig. 3B), between D147 of LHCI-3 and K2/T5 of PsaJ at distances of 3.1 to 3.3 Å (Fig. 3C), between T461 of LHCI-5 and L261 of PsaA at a distance of 3.3 Å (Fig. 3D), between L591 of LHCI-7 and W486 of PsaA at a distance of 3.4 Å (Fig. 3E), between S259 of LHCI-9 and N6 of PsaM at distances of 2.9 to 3.4 Å (Fig. 3F), and between R582 of LHCI-10 and S214 of PsaB at a distance of 3.1 Å (Fig. 3G). The other LHCI subunits are located too far from the PSI core to engage in direct interactions with PSI subunits but instead contribute to the formation of the PSI-LHCI supercomplex through interactions with adjacent LHCI subunits.

LHCI-3 engages in unique pigment-pigment and pigment-protein interactions with PsaJ (Fig. 3, H and I). The side chain of L21 within PsaJ is positioned 3.7 Å from the head group of Ddx724 within LHCI-3. In addition, the conjugated double bond of Ddx724 lies within 3.9 Å of the chlorin ring of Chl a101 within PsaJ. Chl a101 in turn interacts with the chlorin ring of Chl a720 within LHCI-3 at a distance of 3.9 Å. This interaction appears to be predominantly driven by Ddx724 of LHCI-3, which is uniquely positioned among the 13 LHCI subunits to accommodate a Ddx molecule at this site (fig. S4). These findings indicate that the selective association between LHCI-3 and PsaJ is facilitated by the presence of Ddx724.

Possible excitation energy transfer pathways were proposed on the basis of close physical interactions among Chls, specifically between LHCI-1 and PsaB (a), LHCI-3 and PsaJ (b), LHCI-4 and PsaA (c), LHCI-5 and PsaA (d), LHCI-6 and PsaA (e), LHCI-7 and PsaA (f), LHCI-8 and PsaB (g), and LHCI-10 and PsaB (h) (fig. S5).

### Low-energy Chls in the *E. gracilis* PSI-LHCI

It is well established that PSI-LHCI supercomplexes harbor low-energy Chls, typically identified by fluorescence-emission spectra recorded at 77 K. The fluorescence-emission spectrum of *Euglena* PSI-LHCI exhibited a prominent emission peak at 732 nm (*30*). Such low-energy Chls are often associated with oligomeric arrangements, including dimer and trimer formation (*34*–*36*), which exhibit lower energy levels than monomeric Chls, with trimers being more red shifted than dimers. This red shift originates from a head-to-tail (serial or slipped) arrangement of Chls, characteristic of J-aggregates, in which excitonic coupling yields the lowest-energy exciton (*37*).

In the *Euglena* PSI-LHCI structure, we identified a well-ordered cluster of three Chls (Chl a815 of PsaA, Chl a817 of PsaA, and Chl a718 of LHCI-4) at the interface between PsaA and LHCI-4 (Fig. 3J). These Chls adopt similar orientations with inter-Chl distances of 8.4 to 9.3 Å (Fig. 3K). This triple-stacked arrangement closely resembles the Low2 site previously identified in cyanobacterial PSI cores (*35*, *36*) and is likely responsible for the long-wavelength fluorescence observed around 730 nm at 77 K (*36*). Thus, the triple-stacked PsaA-a815/PsaA-a817/LHCI-4-a718 Chls in the *Euglena* PSI-LHCI structure likely represent the lowest-energy Chls.

The chlorin ring of PsaA-a815 is located 3.4 Å from the conjugated double bonds of PsaA-BCR862 (Fig. 3K). This close interaction may function to mediate excitation-energy transfer from PsaA-BCR862 to PsaA-a815/PsaA-a817/LHCI-4-a718, where low-energy Chls may transiently trap the excitation as intermediate energy pools and facilitate its directed transfer to the reaction center via thermally assisted uphill transfer to P700 (*38*, *39*). Alternatively, the Chl-Car interaction may also facilitate excitation-energy quenching through both singlet-singlet and triplet-triplet energy transfer mechanisms (*40*, *41*), thereby contributing to photoprotection under excess light conditions. These observations suggest that the PsaA-a815/PsaA-a817/LHCI-4-a718 triad contributes to excitation-energy transfer and/or quenching in the *Euglena* PSI-LHCI.

### Phylogenetic analysis of PSI-core subunits from *E. gracilis*

**Fig. 4.**
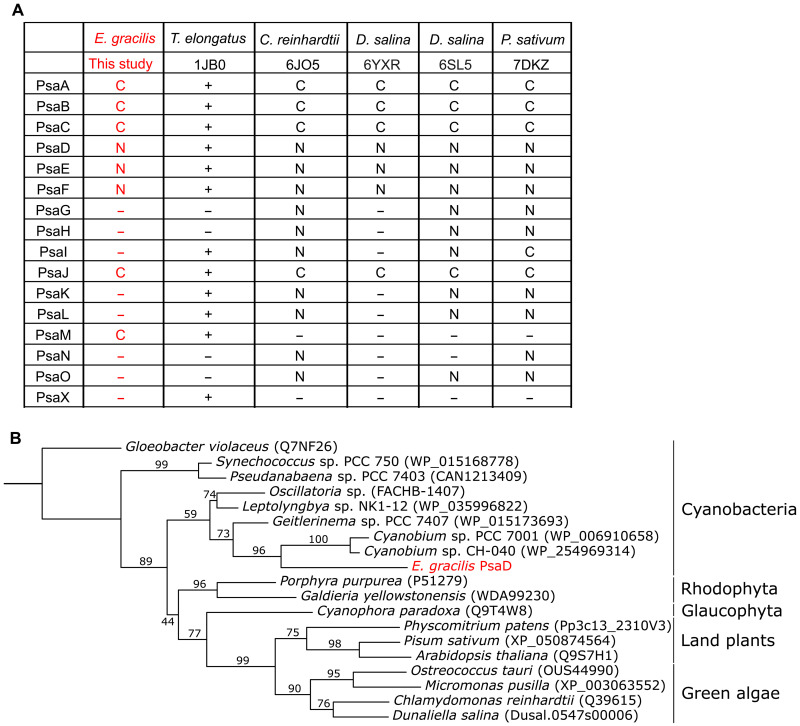
Characteristics of the *Euglena* PSI-core subunits. (**A**) Comparison of PSI subunits that are registered in PDB 1JB0, 6JO5, 6YXR, 6SL5, and 7DKZ. “C” represents chloroplast-encoded subunits, while “N” represents nuclear-encoded subunits. Because cyanobacteria do not have a chloroplast genome, “+” is used when the corresponding core subunits are present. *T. elongatus*, *Thermosynechococcus elongatus*. (**B**) Phylogenetic tree of PsaD. ML tree including *E. gracilis* PsaD and its closest homologs from cyanobacteria, together with representative sequences from glaucophytes, rhodophytes, green algae, and land plants. Ultrafast bootstrap values (1000 replicates) are indicated at the branches.

Among the PSI subunits identified in this study, three subunits—PsaD, PsaE, and PsaF—are encoded by the nuclear genome. The presence of only three nuclear-encoded PSI subunits is rare compared to other green algae (Fig. 4A), but this result is consistent with a previous study (*20*). To investigate the evolutionary origin of these subunits, we examined the sequences of species with high homology to *E. gracilis* subunits using NCBI (National Center for Biotechnology Information) BLAST (Basic Local Alignment Search Tool). PsaE showed the highest homology with the green alga *Monoraphidium neglectum* (score: 103; *E*-value: 1 × 10^−26^), while PsaF showed the highest homology with the green alga *Trebouxia* sp. C0004 (score: 217; *E*-value: 1 × 10^−67^). These results suggest that the two subunits are acquired from green algae.

In contrast to PsaE and PsaF, PsaD showed the highest homology with the cyanobacterium *Geitlerinema* sp. PCC 7407 (score: 208; *E*-value: 2 × 10^−65^). To verify the unexpected result, we constructed a phylogenetic tree for PsaD including orthologs from cyanobacteria, rhodophytes, glaucophytes, green algae, and land plants (Fig. 4B). PsaD of *E. gracilis* was placed within the cyanobacterial clade, not the green algal clade. This indicates that *E. gracilis* PsaD is not of green algal origin but is likely acquired from cyanobacteria via HGT. Notably, this nuclear-encoded PsaD exhibits a distinct evolutionary origin from other nuclear-encoded PSI subunits, highlighting that not only the LHCI subunits but also the core subunits of PSI in *E. gracilis* follow a mosaic evolutionary trajectory.

### Phylogenetic analysis of *Euglena* LHCIs

**Fig. 5.**
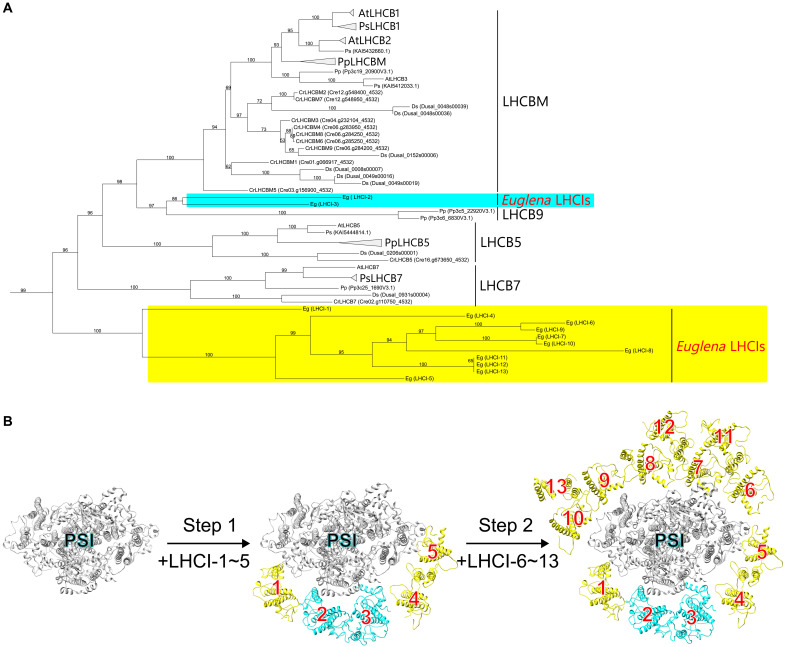
Distribution of the *Euglena* LHCI proteins. (**A**) A maximum-likelihood (ML) tree including the *E. gracilis* LHCI proteins (LHCI-1 to LHCI-13), which are highlighted in cyan and yellow, was constructed using IQ-TREE. For clarity, a portion of the ML tree shown in fig. S6 is excerpted and enlarged here. Ultrafast bootstrap values (1000 replicates) are indicated at the branches. The LHC nomenclature follows our recent study (*88*). At, *A. thaliana*; Ps, *P. sativum*; Pp, *P. patens*; Cr, *C. reinhardtii*; Ds, *D. salina*; Eg, *E. gracilis*. (**B**) Two steps of LHCI assembly in the *Euglena* PSI-LHCI. The numbers 1 to 13 correspond to LHCI-1 to LHCI-13, respectively. The LHCI subunits highlighted in cyan and yellow correspond to the respective clades indicated by the same colors in (A).

We also conducted phylogenetic analysis of the LHC proteins bound to PSI on the basis of the structural analysis (Fig. 5 and figs. S6 and S7). The *E. gracilis* LHCIs formed two major clades (cyan and yellow in Fig. 5A), supporting the previous study (*22*). One of the *E. gracilis* LHC clades, including LHCI-2 and LHCI-3 (cyan), which was previously classified as LHCBM/LHCB1–3 (*22*), was more closely related to LHCB9 of *Physcomitrium patens* (formerly *Physcomitrella patens*) than LHCBM of green algae and land plants. Given that LHCB9 is thought to be a *P. patens*–specific LHC that controls PSI antenna size (*42*–*45*), our result was completely unexpected. In addition, our phylogenetic analysis also revealed that the other *E. gracilis* LHC clade (yellow), previously grouped with LHCB7 (also known as LHCQ) (*22*), diverged earlier than LHCB7. These observations, despite minor differences in tree topology, are virtually identical to the previous studies (*22*, *23*), suggesting that the LHC proteins bound to PSI in *E. gracilis* are derived from at least two green algal LHCII proteins and have subsequently undergone lineage-specific diversification and rearrangement.

The two LHCI clades were mapped onto the PSI-LHCI structure (fig. S8). LHCI-2 and LHCI-3 closely related to LHCB9 of *P. patens* (cyan in fig. S8) are positioned on the PsaF side (Fig. 1). In the other clade of LHCIs (yellow in fig. S8), the early diverging members—LHCI-1, LHCI-4, and LHCI-5—also associate with the PsaF side, whereas the remaining members bind on the opposite side (Fig. 1). These results suggest that both the phylogenetically distinct LHCI clade related to LHCB9 and the early diverging members of the other clade may have undergone evolutionary optimization to occupy structurally and functionally advantageous LHCI positions for efficient and stable excitation-energy transfer.

## DISCUSSION

### Molecular evolution of the *E. gracilis* PSI-core subunits

In this study, we elucidated the structure of *E. gracilis* PSI-LHCI and revealed its evolutionary significance. Our structural analysis confirmed that three nuclear-encoded subunits (PsaD, PsaE, and PsaF) and five subunits encoded by the chloroplast genome (PsaA, PsaB, PsaC, PsaJ, and PsaM) are successfully incorporated into the PSI core. Compared with the model green alga *C. reinhardtii* PSI, the *E. gracilis* PSI lacks seven nuclear-encoded subunits (PsaG, PsaH, PsaI, PsaK, PsaL, PsaN, and PsaO), resulting in a markedly reduced PSI configuration. This distinctive feature sets the stage for exploring the unique evolutionary trajectory of Euglenophyceae PSI, as detailed below.

Among the chloroplast-encoded subunits, PsaA, PsaB, PsaC, and PsaJ show high conservation across Euglenophyceae, reflecting their fundamental importance in the PSI function. In contrast, PsaI and PsaM exhibit remarkable diversity within this group. While *E. gracilis* retains PsaM but lacks PsaI, *Eutreptia viridis*, a member of Euglenophyceae, exhibits the reverse pattern, encoding PsaI but not PsaM (*46*). Other Euglenophyceae species such as *Strombomonas acuminata* and *Colacium vesiculosum* have both PSI proteins (*47*). *P. parkeae*, which shares a common ancestor with the presumed endosymbiotic origin of *Euglena* chloroplast, encodes both PsaI and PsaM in its chloroplast genome (*15*). This phylogenetic evidence strongly suggests that the absence of PsaI in *E. gracilis* reflects a secondary loss during Euglenophyceae evolution, rather than being inherited from the endosymbiotic ancestor.

The nuclear-encoded subunits indicate an even more complex evolutionary history. While PsaE and PsaF likely originate from green algae (*20*), our phylogenetic analysis showed that PsaD originated from cyanobacteria via HGT (Fig. 4B). This mosaic origin of PSI subunits—combining green algal EGT/HGT and cyanobacterial HGT—highlights the intricate evolutionary processes that shaped the *E. gracilis* photosynthetic apparatus. The integration of PsaD from such a distant source underscores the capacity of *E. gracilis* to assimilate foreign genes conferring selective advantages. For PsaE and PsaF, acquisition via HGT from other green algae cannot be excluded, alongside EGT from Euglenophyceae. However, a definitive conclusion cannot be drawn owing to the scarcity of prasinophyte genomes and the short length of these proteins. Resolving their origin will require further advances in prasinophyte genomics.

Despite this demonstrated capacity for gene acquisition, the PSI of *E. gracilis* has evolved into a minimal configuration lacking six peripheral subunits present in other green algae (Fig. 1A). This simplification likely reflects the inherent difficulty of establishing functional, nuclear-encoded, chloroplast-targeted proteins via EGT or HGT, which requires not only the initial gene transfer but also the subsequent evolution of regulatory sequence motifs, such as promoters and targeting signals (*48*). These stringent demands probably constrained subunit incorporation to those indispensable for PSI activity, giving rise to the streamlined architecture observed in *E. gracilis*.

Our structural analysis further revealed that the PSI core comprises only eight subunits: PsaA, PsaB, PsaC, PsaD, PsaE, PsaF, PsaJ, and PsaM (Fig. 1A). Notably, the *D. salina* PSI-LHCI shows a similarly reduced composition with seven subunits (*33*). The convergent emergence of such pared-down architectures in phylogenetically distant organisms—*Euglena* and *Dunaliella* (Chlorophyta)—suggests that this reduced configuration represents the functional minimum of PSI activity. The absence of subunits generally regarded as conserved across cyanobacteria, green algae, and land plants, including PsaI, PsaK, and PsaL, challenges current definitions of PSI essentiality. To fully understand the evolutionary and functional implications of these streamlined PSI forms, further structural and functional studies across diverse photosynthetic lineages are required.

### Molecular evolution of *E. gracilis* LHCIs

LHCs of *Euglena* are derived from green algal LHCs but have followed a distinctive evolutionary trajectory (*22*, *23*). A notable feature is that many *Euglena* LHCs are transcribed and translated as polycistrons (*49*), suggesting that after the acquisition of ancestral LHC sequences, duplication and recombination events produced tandem arrays of LHC subunits. Consequently, the LHC composition of *Euglena* differs notably from that of green algae, except for CP29. All other *Euglena* LHCs have been reported to be closely related to LHCBM and LHCB7 (*22*). LHCB7 was formerly referred to as LHCQ; however, this nomenclature is no longer used, as LHCQ now designates a distinct LHC subfamily specific to the red lineage (*50*). This phylogenetic pattern suggests that only a limited set of green algal LHC genes was acquired during endosymbiosis and subsequently diversified to form functional photosystem supercomplexes. Nevertheless, in the absence of structural data, it has remained unclear which LHCs bind to specific positions in PSI and PSII.

Our phylogenetic analysis revealed that the LHCIs of *E. gracilis* fall into two groups: one closely related to LHCBM/LHCB and the other to LHCB7 (Fig. 5 and figs. S6 and S7). Because LHCBM and LHCB are associated with PSII in green algae and land plants, respectively, we infer that their last common ancestor had LHCBM/LHCB–type LHCs functioning with PSII but not with PSI. Notably, these *E. gracilis* LHCs branched from the LHCII clade rather than the LHCI clade, indicating their evolutionary origin from PSII-associated LHCs. This indicates that the light-harvesting system within the *E. gracilis* PSI-LHCI is completely different from that of green algae and other photosynthetic organisms and is uniquely reconstructed in *Euglena* on the basis of at least two types of LHCs that have been recruited from the LHCII lineage. Based on the phylogenetic tree in this study, the former is more closely related to LHCB9 of *P. patens* than LHCBM, while the latter branched earlier than LHCB7. LHCB9 is a specific LHC found in *P. patens*, known to bind to PSI and regulate the enlargement of PSI antenna size (*42*–*45*). The formation of a clade between one group of *E. gracilis* LHCs and the *P*. *patens*-specific LHCB9 was an unexpected result. This suggests the possibility that *E. gracilis* acquired *P*. *patens* LHCB9 through HGT or that ancestral LHCB9 sequences existed in green algae.

### Prerequisite for LHCI belt formation

In most green-lineage organisms, one or two LHCI belts are positioned on the PsaF side of the PSI core, each consisting of four LHCI subunits arranged as two heterodimers (*9*–*13*). The N and C termini of the LHCI subunits are thought to contribute to the structural stabilization of each heterodimer, as previously suggested (*51*). Notably, these characteristic terminal motifs are absent in LHCBM/LHCB proteins comprising LHCII (*51*). In addition, distinct structural differences in loop regions and transmembrane helices have been proposed between LHCA and LHCBM/LHCB proteins (*52*), preventing the formation of such heterodimers by LHCII proteins.

The *Euglena* PSI-LHCI structure revealed the absence of typical LHCI belts at the side of PsaF and PsaJ (Fig. 1). Our phylogenetic analysis showed that the *Euglena* LHCI proteins are placed within the green-lineage LHCII clade, rather than clustering with the canonical LHCI clade of the green lineage (Fig. 5 and figs. S6 and S7). By contrast, the composition of the PSI core does not appear to influence LHCI belt formation, as a well-defined LHCI belt is assembled even in the minimal PSI core of *D. salina* (Fig. 2C) (*33*). These findings indicate that the membership in the canonical LHCA set of the green lineage is a prerequisite for LHCI belt formation, regardless of PSI core composition. Once genomic turbulence such as LHC gene loss occurs during endosymbiosis, LHCI must be reorganized through gene duplication and neolocalization to reconstruct the belt with an altered subunit composition. Accordingly, only those restructured supercomplexes that successfully reassembled an LHCI belt have persisted and are observable today.

### Toward understanding of *Euglena* LHCI binding modes

In contrast to the orderly heterodimeric arrangement of LHCI subunits observed in well-characterized green-lineage organisms, the LHCI configuration in *E. gracilis* tends to resemble that of red-lineage organisms, appearing structurally less constrained and more variable (*10*). Phylogenetic analysis revealed that the LHCR subfamily, which includes red algal LHCI proteins, diverged with high support from the clades comprising the LHCA and LHCBM/LHCB subfamilies, which correspond to LHCI and LHCII, respectively, in conventional green-lineage organisms (figs. S6 and S7). This further reinforces the idea that only LHCA proteins originating from typical green-lineage organisms can form heterodimers and assemble into an LHCI belt.

It is important to note that the LHCI binding mode in red-lineage organisms appears to exhibit a certain degree of interaction selectivity, as revealed by the structural comparison of PSI-LHCI supercomplexes, called PSI-FCPI (PSI-fucoxanthin Chl *a*/*c*–binding protein I), between the diatoms *Chaetoceros gracilis* and *Thalassiosira pseudonana* (*53*). This level of insight is only attainable through comprehensive genome sequencing combined with high-quality phylogenetic and structural analyses of PSI-LHCI. In *E. gracilis*, the nuclear-encoded sequences of PSI and LHCI proteins were not derived from genome sequencing. Once the complete genome of *E. gracilis* becomes available, comparative analysis of LHCI binding modes—with organisms in which LHCI subunits belong to the canonical green-lineage LHCII group, such as *E. gracilis*—may unveil specific molecular mechanisms underlying the selective association of LHCIs with defined positions in the PSI-LHCI supercomplex.

### Selective binding of Car molecules to LHCs

The *Euglena* LHCI subunits bind Ddx molecules (fig. S4). This structure provides the first evidence that Ddx, instead of lutein, neoxanthin, and violaxanthin, is naturally bound to green-lineage LHCIs, whereas its association with red-lineage LHCIs has been well established (*10*). Previous in vivo and in vitro studies suggested that Cars can be replaced in LHCs (*54*–*57*), implying that while specific structural constraints exist at each binding pocket, complete specificity is not enforced. Thus, *Euglena* may have come to preferentially incorporate Ddx into its LHCs because of its predominant expression during evolution. Although not all components of the Ddx biosynthetic pathway in *Euglena* can be attributed to red-lineage origins, at least some enzymes, such as CYP97F, clearly derive from red-lineage secondary endosymbiotic algae (*58*–*60*). The presence of such heterologously acquired Cars in *Euglena* LHCI highlights its evolution through complex gene transfer events beyond the green lineage. Therefore, *Euglena* represents an evolutionary mosaic in terms of Cars using the heterologously acquired Ddx for light harvesting in LHCIs.

### Assembly model of *Euglena* LHCI

In most green-lineage organisms, the formation of an LHCI inner belt on the PsaF side is an evolutionarily conserved feature, reflecting its pivotal role in excitation-energy transfer from LHCI to PSI. The absence of such a belt in *E. gracilis* suggests the adoption of an alternative strategy for LHCI assembly. On the basis of these findings, we propose a schematic model of the assembly of *Euglena* LHCI (Fig. 5B). Considering the importance of the PsaF side in LHCI binding among typical green-lineage organisms, LHCI subunits with key functional roles are likely to assemble preferentially at this site (step 1 in Fig. 5B). Among the LHCI clade closely related to LHCB9 (cyan in Fig. 5A), LHCI-3 binds to PsaJ through D147, Chl a720, and Ddx724, each contributing to the specific pigment-protein and protein-protein interactions that facilitate recognition (Fig. 3, C, H, and I). LHCI-2 lacks any detectable protein-protein interactions with PSI, suggesting that its association with PSI occurs through cobinding with LHCI-3. In parallel, the early diverging members in the other LHCI clade of *Euglena* (yellow in Fig. 5A) initially occupy the PsaF side. While LHCI-1 and LHCI-5 directly interact with PSI (Fig. 3, B and D), LHCI-4 does not exhibit such interactions and is therefore presumed to associate with PSI via LHCI-5. Following the establishment of LHCI assembly at the PsaF side, the remaining LHCI subunits, LHCI-6 to LHCI-13, likely bind to the opposite side of PSI (step 2 in Fig. 5B).

The functional significance of this assembly model likely lies in the optimization of excitation-energy transfer to P700. On the PsaF side, close physical proximity between Chls of LHCI and PSI was identified for LHCI-1, LHCI-3, LHCI-4, and LHCI-5 (arrows a to d in fig. S5). Among these, the shortest distance between the PSI side acceptor Chl and P700 was found to be 39.7 Å between Chl a101 of PsaJ and P700 (fig. S9), suggesting that excitation energy from LHCI-3 may be efficiently relayed to P700 via PsaJ. It is noteworthy that in the PSI-LHCI structures of *P. sativum* and *D. salina*, LHCIs located at the sites of Ps1 (Ds1) and Ps4 (Ds4) (Fig. 2, B and C) appear to participate in excitation-energy transfer to PSI, consistent with their spatial proximity to PSI-bound Chls. In contrast, LHCIs at the Ps2 (Ds2) and Ps3 (Ds3) sites (Fig. 2B, C) are located further away from PSI-bound Chls, making them less likely to function as direct energy donors. These observations suggest that *Euglena*, which lacks the canonical LHCA subfamily (Fig. 5A and figs. S6 and S7), evolved a configuration that circumvents the structural constraints imposed by the LHCI belt, bringing antenna Chls into closer proximity with P700. This architectural arrangement may be the outcome of evolutionary optimization to enhance both the efficiency and stability of excitation-energy transfer between LHCI and PSI.

### Divergent evolution of PSI-LHCI in *Euglena*—Beyond the typical green lineage

**Fig. 6.**
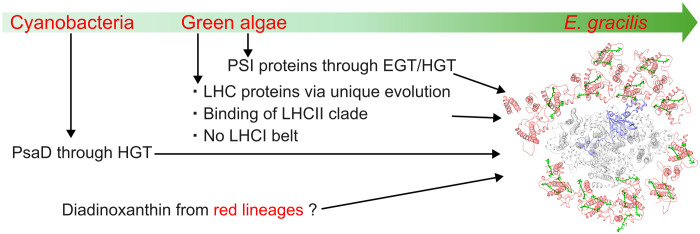
Evolutionary scheme of the *Euglena* PSI-LHCI. A proposed evolutionary trajectory of the PSI-LHCI of *E. gracilis* is illustrated, with a green arrow indicating its origin from cyanobacteria through green algae. The PSI-LHCI structure is shown from the stromal side, with subunits and pigments colored as follows: PsaD in blue, other PSI core subunits in gray, LHCI proteins in red, and Ddx molecules in green. Black arrows highlight key evolutionary events specific to the *Euglena* PSI-LHCI. The PsaD subunit was inherited directly from cyanobacteria, whereas the remaining PSI subunits were acquired from green algae. The LHCI proteins also originated from green algal LHCs but subsequently underwent unique evolutionary modifications. Notably, the LHCI subunits in *Euglena* do not assemble into the characteristic belt structure found in green-lineage organisms and exhibit sequence similarity to LHCII proteins. Ddxs are incorporated in the structure, although the evolutionary origin of their biosynthetic pathway may trace back to red-lineage organisms.

This study demonstrates the four distinguishing features of the *Euglena* PSI-LHCI structure (Fig. 6): (i) the presence of PsaD that is closely related to its cyanobacterial counterpart instead of the green-lineage species, (ii) the presence of LHCI subunits with sequence similarity to conventional green-lineage LHCII proteins, (iii) the incorporation of the red-lineage Car Ddx, and (iv) the absence of a typical LHCI belt structure commonly found in well-characterized green-lineage organisms. These features suggest that the *Euglena* PSI-LHCI does not retain the characteristics of the vertically inherited PSI-LHCI found in green-lineage organisms but instead reflects a distinct evolutionary trajectory shaped by endosymbiosis and HGT. The functional and evolutionary significance of such an atypical light-harvesting system—one that aligns with neither green nor red lineages—has only recently begun to attract attention. As further structural and phylogenetic insights are obtained from organisms that defy existing evolutionary classifications, this recently raised question may be solved ultimately.

## MATERIALS AND METHODS

### Cryo-EM data collection

The PSI-LHCI supercomplexes from *E. gracilis* strain Z were purified, and their biochemical and spectroscopic properties have been previously characterized (*30*). Quantifoil grids (R1.2/1.3, Cu, 300 mesh) were glow discharged for 10 s using a JEOL JEC-3000FC at a vacuum of 0.7 Pa and a discharge current of 10 mA. A 3-μl aliquot of the *E. gracilis* PSI-LHCI supercomplex (2.5 mg of Chl ml^−1^) in 20 mM MES-NaOH (pH 6.5) buffer containing 10 mM NaCl and 0.03% sucrose monolaurate was then applied to the grids in an FEI Vitrobot Mark IV (Thermo Fisher Scientific). Grids were blotted for 4 s with filter papers at 8°C under 100% humidity and subsequently plunged into liquid ethane cooled by liquid nitrogen. The frozen grid was transferred into a CRYO ARM 300 electron microscope (JEOL) equipped with a cold-field emission gun operated at 300 kV. All image stacks were collected from 5 by 5 holes per stage adjustment to the central hole, and image shifts were applied to the surrounding holes while maintaining an axial coma–free condition. The images were recorded with an in-column energy filter with a slit width of 20 eV and at a nominal magnification of ×60,000 on a direct electron detector (Gatan K3, AMETEK). The nominal defocus range was −1.8 to −1.2 μm. The physical pixel size corresponded to 0.752 Å. Each image stack was exposed at a dose rate of 23.46 e^−^ Å^−2^ s^−1^ for 2.13 s in correlated-double sampling mode with 50 dose-fractionated movie frames. In total, 7575 image stacks were collected.

### Cryo-EM image processing

The movie frames were aligned and dose weighted using MotionCor2 (*61*), and the contrast transfer function was estimated with CTFFIND4 (*62*). Subsequent processing was carried out in RELION-4.0 (*63*). An initial set of 100 micrographs was subjected to Laplacian-of-Gaussian blob picking with a particle diameter range of 120 to 250 Å. Following particle extraction, 2D classification revealed well-defined PSI-LHCI features, and the selected classes were then used as templates for particle picking across the entire dataset. A total of 1,317,040 particles were automatically picked and subjected to two rounds of 2D classification. From these, 507,917 particles belonging to well-defined classes were retained and further refined through three rounds of 3D classification without imposing symmetry. An initial model for the first 3D classification was generated de novo from 2D classification. As shown in fig. S1C, the final PSI-LHCI structure was reconstructed from 37,800 particles. The overall resolution of the cryo-EM map was estimated to be 2.82 Å by the gold-standard Fourier shell correlation curve with a cutoff value of 0.143 (fig. S2A) (*64*). Local resolution (fig. S2C) was calculated in RELION-4.0 using its built-in local-resolution routine (relion_postprocess).

### Model building and refinement

The amino acid sequences of LHCs were retrieved from three independently published transcriptome assemblies of *E. gracilis* (*16*, *17*, *28*), which together contained 38, 40, and 57 LHCs. Open reading frames were predicted and translated with TransDecoder version 5.5.0 (https://github.com/TransDecoder/TransDecoder). LHC and PSI proteins were identified by BLASTp (Protein BLAST) version 2.13.0+ searches using *C. reinhardtii* PSI and LHC sequences as queries. Redundant identical sequences were removed using CD-HIT. The plastid-encoded subunits PsaA, PsaB, PsaC, PsaJ, and PsaM were obtained from the plastid genome (NC_001603.2), whereas PsaD, PsaE, and PsaF were identified only in the transcriptome datasets and are nuclear-encoded genes.

Two types of the cryo-EM maps were used for the model building of the PSI-LHCI supercomplex: One was a postprocessed map, and the other was a denoised map using Topaz version 0.2.4 (*65*). The postprocessed map was denoised using the trained model in 100 epochs with two half-maps. Initial models of each subunit in the PSI-LHCI supercomplex were generated using the transcriptome-derived amino acid sequences as input for the automated model-building program ModelAngelo (*29*). The resulting models were subsequently inspected and manually validated against the cryo-EM maps in Coot to confirm that they showed no discrepancies (*66*). Each model was built on the basis of interpretable features from the density maps at contour levels of 2.0 and 2.5 σ in the denoised and postprocessed maps, respectively. For the assignment of Chls, because of the relatively low resolution of the map, all Chls were modeled as Chl *a*. For the assignment of Cars, the same rationale was applied: Cars in the PSI core and LHCIs were modeled as BCR and Ddx, respectively. The PSI-LHCI structure was refined with phenix.real_space_refine (*67*) and Servalcat (*68*) with geometric restraints for the protein-cofactor coordination. The final model was validated with MolProbity (*69*), EMRinger (*70*), and *Q*-score (*71*). The statistics for all data collection and structure refinement are summarized in tables S1 and S2. All structural figures were made by PyMOL (*72*), UCSF Chimera (*73*), and UCSF ChimeraX (*74*).

Given that the numbering of Chls, Cars, and other cofactors in this paper was different from those of the Protein Data Bank (PDB) data, we listed the relationship of their numbering in this paper with that in the PDB data in tables S6 to S9.

### Phylogenetic analysis of LHC proteins

The LHC sequences of *Arabidopsis thaliana* (*75*), *P. patens* (*76*), and *C. reinhardtii* (*77*) were retrieved from the Phytozome database (https://phytozome.jgi.doe.gov/), while those of *Ostreococcus tauri* (*78*), *D. salina* (*79*, *80*), and *Porphyridium purpureum* (*81*) were obtained from the Phycocosm database (https://phycocosm.jgi.doe.gov/). The LHC sequences of *P. sativum* were retrieved from the Pea Genome Database (www.peagdb.com/) (*82*, *83*). The LHCSR sequences from *C. reinhardtii*, *O. tauri*, and *P. patens* were used as outgroups for phylogenetic analysis.

Amino acid sequences of LHCs were aligned using MAFFT (version 7.511) with the L-INS-I option (*84*). The alignment was trimmed using ClipKIT (version 2.1.1) with default parameters (*85*). An ML phylogenetic tree was generated using IQ-TREE (version 2.3.6) under the best-fit model (Q.pfam+F+R5) (*86*). Ultrafast bootstrap values (1000 replicates) are indicated at the branches. The tree was visualized using TreeViewer version 2.2.0 (*87*). Some LHCs were collapsed in the tree, while the uncollapsed tree is also shown (fig. S7). The LHC nomenclature follows our previous study (*88*).

### Homology searches of *E. gracilis* PSI proteins and phylogenetic analysis of PsaD

The NCBI BLASTp program was used for homology searches using *Euglena* PsaD, PsaE, and PsaF against nonredundant protein sequences in May 2025. The phylogenetic tree for PsaD protein was constructed like the LHC trees using the closest homologs of *Euglena* proteins supplemented with orthologs from cyanobacteria, glaucophytes, rhodophytes, green algae, and land plants (Fig. 4B). The best-fit model for amino acid substitution determined by IQ-TREE was WAG+I+G4.

## References

[1] R. E. Blankenship, *Molecular Mechanisms of Photosynthesis* (Wiley-Blackwell, ed. 3, 2021).

[2] J. H. Golbeck, Structure and function of photosystem I. Ann. Rev. Plant Physiol. Plant Mol. Biol. 43, 293–324 (1992).

[3] K. Brettel, W. Leibl, Electron transfer in photosystem I. Biochim. Biophys. Acta, Bioenerg. 1507, 100–114 (2001). 10.1016/s0005-2728(01)00202-x 11687210

[4] J.-R. Shen, The structure of photosystem II and the mechanism of water oxidation in photosynthesis. Annu. Rev. Plant Biol. 66, 23–48 (2015). 25746448 10.1146/annurev-arplant-050312-120129

[5] D. Shevela, J. F. Kern, G. Govindjee, J. Messinger, Solar energy conversion by photosystem II: Principles and structures. Photosynth. Res. 156, 279–307 (2023). 36826741 10.1007/s11120-022-00991-y PMC10203033

[6] J. Engelken, H. Brinkmann, I. Adamska, Taxonomic distribution and origins of the extended LHC (light-harvesting complex) antenna protein superfamily. BMC Evol. Biol. 10, 233 (2010). 20673336 10.1186/1471-2148-10-233 PMC3020630

[7] S. Sturm, J. Engelken, A. Gruber, S. Vugrinec, P. G. Kroth, I. Adamska, J. Lavaud, A novel type of light-harvesting antenna protein of red algal origin in algae with secondary plastids. BMC Evol. Biol. 13, 159 (2013). 23899289 10.1186/1471-2148-13-159 PMC3750529

[8] P. G. Falkowski, M. E. Katz, A. H. Knoll, A. Quigg, J. A. Raven, O. Schofield, F. J. R. Taylor, The evolution of modern eukaryotic phytoplankton. Science 305, 354–360 (2004). 15256663 10.1126/science.1095964

[9] M. Hippler, N. Nelson, The plasticity of photosystem I. Plant Cell Physiol. 62, 1073–1081 (2021). 33768246 10.1093/pcp/pcab046

[10] J.-R. Shen, in *Macromolecular Protein Complexes IV. Subcellular Biochemistry,* J. R. Harris, J. Marles-Wright, Eds. (Springer, 2022), vol. 99, pp. 351–377.

[11] N. Nelson, Investigating the balance between structural conservation and functional flexibility in photosystem I. Int. J. Mol. Sci. 25, 5073 (2024). 38791114 10.3390/ijms25105073 PMC11121529

[12] M. Iwai, D. Patel-Tupper, K. K. Niyogi, Structural diversity in eukaryotic photosynthetic light harvesting. Annu. Rev. Plant Biol. 75, 119–152 (2024). 38360524 10.1146/annurev-arplant-070623-015519

[13] X. Pan, P. Cao, X. Su, Z. Liu, M. Li, Structural analysis and comparison of light-harvesting complexes I and II. Biochim. Biophys. Acta Bioenerg. 1861, 148038 (2020). 31229568 10.1016/j.bbabio.2019.06.010

[14] R. B. Hallick, L. Hong, R. G. Drager, M. R. Favreau, A. Monfort, B. Orsat, A. Spielmann, E. Stutz, Complete sequence of *Euglena gracilis* chloroplast DNA. Nucleic Acids Res. 21, 3537–3544 (1993). 8346031 10.1093/nar/21.15.3537 PMC331456

[15] M. Turmel, M.-C. Gagnon, C. J. O'Kelly, C. Otis, C. Lemieux, The chloroplast genomes of the green algae *Pyramimonas*, *Monomastix*, and *Pycnococcus* shed new light on the evolutionary history of prasinophytes and the origin of the secondary chloroplasts of euglenids. Mol. Biol. Evol. 26, 631–648 (2009). 19074760 10.1093/molbev/msn285

[16] J. Cordoba, E. Perez, M. Van Vlierberghe, A. R. Bertrand, V. Lupo, P. Cardol, D. Baurain, De novo transcriptome meta-assembly of the mixotrophic freshwater microalga *Euglena gracilis*. Genes 12, 842 (2021). 34072576 10.3390/genes12060842 PMC8227486

[17] T. G. E. Ebenezer, M. Zoltner, A. Burrell, A. Nenarokova, A. M. G. Novák Vanclová, B. Prasad, P. Soukal, C. Santana-Molina, E. O'Neill, N. N. Nankissoor, N. Vadakedath, V. Daiker, S. Obado, S. Silva-Pereira, A. P. Jackson, D. P. Devos, J. Lukeš, M. Lebert, S. Vaughan, V. Hampl, M. Carrington, M. L. Ginger, J. B. Dacks, S. Kelly, M. C. Field, Transcriptome, proteome and draft genome of *Euglena gracilis*. BMC Biol. 17, 11 (2019). 30732613 10.1186/s12915-019-0626-8 PMC6366073

[18] S. Maruyama, T. Suzaki, A. P. M. Weber, J. M. Archibald, H. Nozaki, Eukaryote-to-eukaryote gene transfer gives rise to genome mosaicism in euglenids. BMC Evol. Biol. 11, 105 (2011). 21501489 10.1186/1471-2148-11-105 PMC3101172

[19] N. Ahmadinejad, T. Dagan, W. Martin, Genome history in the symbiotic hybrid *Euglena gracilis*. Gene 402, 35–39 (2007). 17716833 10.1016/j.gene.2007.07.023

[20] R. Sobotka, H. J. Esson, P. Koník, E. Trsková, L. Moravcová, A. Horák, P. Dufková, M. Oborník, Extensive gain and loss of photosystem I subunits in chromerid algae, photosynthetic relatives of apicomplexans. Sci. Rep. 7, 13214 (2017). 29038514 10.1038/s41598-017-13575-x PMC5643376

[21] D. G. Durnford, M. W. Gray, Analysis of *Euglena gracilis* plastid-targeted proteins reveals different classes of transit sequences. Eukaryot. Cell 5, 2079–2091 (2006). 16998072 10.1128/EC.00222-06 PMC1694827

[22] A. G. Koziol, T. Borza, K.-I. Ishida, P. Keeling, R. W. Lee, D. G. Durnford, Tracing the evolution of the light-harvesting antennae in chlorophyll *a*/*b*-containing organisms. Plant Physiol. 143, 1802–1816 (2007). 17307901 10.1104/pp.106.092536 PMC1851817

[23] A. G. Koziol, D. G. Durnford, *Euglena* light-harvesting complexes are encoded by multifarious polyprotein mRNAs that evolve in concert. Mol. Biol. Evol. 25, 92–100 (2008). 17947344 10.1093/molbev/msm232

[24] K. Kato, T. Hamaguchi, M. Kumazawa, Y. Nakajima, K. Ifuku, S. Hirooka, Y. Hirose, S.-y. Miyagishima, T. Suzuki, K. Kawakami, N. Dohmae, K. Yonekura, J.-R. Shen, R. Nagao, The structure of PSI-LHCI from *Cyanidium caldarium* provides evolutionary insights into conservation and diversity of red-lineage LHCs. Proc. Natl. Acad. Sci. U.S.A. 121, e2319658121 (2024). 38442179 10.1073/pnas.2319658121 PMC10945839

[25] M. Kumazawa, K. Ifuku, Unraveling the evolutionary trajectory of LHCI in red-lineage algae: Conservation, diversification, and neolocalization. iScience 27, 110897 (2024). 39386759 10.1016/j.isci.2024.110897 PMC11462038

[26] K. Kato, M. Kumazawa, Y. Nakajima, T. Suzuki, N. Dohmae, J.-R. Shen, K. Ifuku, R. Nagao, Structure of a photosystem I supercomplex from *Galdieria sulphuraria* close to an ancestral red alga. Sci. Adv. 11, eadv7488 (2025). 40378202 10.1126/sciadv.adv7488 PMC12083527

[27] Y. Mazor, A. Borovikova, N. Nelson, The structure of plant photosystem I super-complex at 2.8 Å resolution. eLife 4, e07433 (2015). 10.7554/eLife.07433 PMC4487076 26076232

[28] Y. Yoshida, T. Tomiyama, T. Maruta, M. Tomita, T. Ishikawa, K. Arakawa, De novo assembly and comparative transcriptome analysis of *Euglena gracilis* in response to anaerobic conditions. BMC Genomics 17, 182 (2016). 26939900 10.1186/s12864-016-2540-6 PMC4778363

[29] K. Jamali, L. Käll, R. Zhang, A. Brown, D. Kimanius, S. H. W. Scheres, Automated model building and protein identification in cryo-EM maps. Nature 628, 450–457 (2024). 38408488 10.1038/s41586-024-07215-4 PMC11006616

[30] R. Sakamoto, K. Kato, Y. Nakajima, J.-R. Shen, R. Nagao, Purification and pigment analysis of diadinoxanthin-binding PSI-LHCI supercomplexes from *Euglena gracilis* strain Z. microPubl. Biol. 10.17912/micropub.biology.001678 (2025). 10.17912/micropub.biology.001678 PMC12364551 40838119

[31] A. Ben-Shem, F. Frolow, N. Nelson, Crystal structure of plant photosystem I. Nature 426, 630–635 (2003). 14668855 10.1038/nature02200

[32] J. Wang, L.-J. Yu, W. Wang, Q. Yan, T. Kuang, X. Qin, J.-R. Shen, Structure of plant photosystem I-light harvesting complex I supercomplex at 2.4 Å resolution. J. Integr. Plant Biol. 63, 1367–1381 (2021). 33788400 10.1111/jipb.13095

[33] A. Perez-Boerema, D. Klaiman, I. Caspy, S. Y. Netzer-El, A. Amunts, N. Nelson, Structure of a minimal photosystem I from the green alga *Dunaliella salina*. Nat. Plants 6, 321–327 (2020). 32123351 10.1038/s41477-020-0611-9

[34] E. Schlodder, V. V. Shubin, E. El-Mohsnawy, M. Roegner, N. V. Karapetyan, Steady-state and transient polarized absorption spectroscopy of photosystem I complexes from the cyanobacteria *Arthrospira platensis* and *Thermosynechococcus elongatus*. Biochim. Biophys. Acta, Bioenerg. 1767, 732–741 (2007). 10.1016/j.bbabio.2007.01.013 17321489

[35] H. Toporik, A. Khmelnitskiy, Z. Dobson, R. Riddle, D. Williams, S. Lin, R. Jankowiak, Y. Mazor, The structure of a red-shifted photosystem I reveals a red site in the core antenna. Nat. Commun. 11, 5279 (2020). 33077842 10.1038/s41467-020-18884-w PMC7573975

[36] K. Kato, T. Hamaguchi, R. Nagao, K. Kawakami, Y. Ueno, T. Suzuki, H. Uchida, A. Murakami, Y. Nakajima, M. Yokono, S. Akimoto, N. Dohmae, K. Yonekura, J.-R. Shen, Structural basis for the absence of low-energy chlorophylls in a photosystem I trimer from *Gloeobacter violaceus*. eLife 11, e73990 (2022). 35404232 10.7554/eLife.73990 PMC9000952

[37] J. Parkash, J. H. Robblee, J. Agnew, E. Gibbs, P. Collings, R. F. Pasternack, J. C. de Paula, Depolarized resonance light scattering by porphyrin and chlorophyll *a* aggregates. Biophys. J. 74, 2089–2099 (1998). 9545068 10.1016/S0006-3495(98)77916-0 PMC1299550

[38] B. Gobets, R. van Grondelle, Energy transfer and trapping in photosystem I. Biochim. Biophys. Acta, Bioenerg. 1507, 80–99 (2001). 10.1016/s0005-2728(01)00203-1 11687209

[39] B. Gobets, I. H. M. van Stokkum, M. Rögner, J. Kruip, E. Schlodder, N. V. Karapetyan, J. P. Dekker, R. van Grondelle, Time-resolved fluorescence emission measurements of photosystem I particles of various cyanobacteria: A unified compartmental model. Biophys. J. 81, 407–424 (2001). 11423424 10.1016/S0006-3495(01)75709-8 PMC1301521

[40] T. Polívka, V. Sundström, Ultrafast dynamics of carotenoid excited states−From solution to natural and artificial systems. Chem. Rev. 104, 2021–2072 (2004). 15080720 10.1021/cr020674n

[41] R. Bassi, L. Dall'Osto, Dissipation of light energy absorbed in excess: The molecular mechanisms. Annu. Rev. Plant Biol. 72, 47–76 (2021). 34143647 10.1146/annurev-arplant-071720-015522

[42] M. Iwai, M. Yokono, M. Kono, K. Noguchi, S. Akimoto, A. Nakano, Light-harvesting complex Lhcb9 confers a green alga-type photosystem I supercomplex to the moss *Physcomitrella patens*. Nat. Plants 1, 14008 (2015). 27246756 10.1038/nplants.2014.8

[43] M. Iwai, P. Grob, A. T. Iavarone, E. Nogales, K. K. Niyogi, A unique supramolecular organization of photosystem I in the moss *Physcomitrella patens*. Nat. Plants 4, 904–909 (2018). 30374090 10.1038/s41477-018-0271-1 PMC7806276

[44] A. Pinnola, A. Alboresi, L. Nosek, D. Semchonok, A. Rameez, A. Trotta, F. Barozzi, R. Kouřil, L. Dall'Osto, E.-M. Aro, E. J. Boekema, R. Bassi, A LHCB9-dependent photosystem I megacomplex induced under low light in *Physcomitrella patens*. Nat. Plants 4, 910–919 (2018). 30374091 10.1038/s41477-018-0270-2

[45] S. Zhang, K. Tang, Q. Yan, X. Li, L. Shen, W. Wang, Y.-K. He, T. Kuang, G. Han, J.-R. Shen, X. Zhang, Structural insights into a unique PSI-LHCI-LHCII-Lhcb9 supercomplex from moss *Physcomitrium patens*. Nat. Plants 9, 832–846 (2023). 37095225 10.1038/s41477-023-01401-4

[46] K. E. Wiegert, M. S. Bennett, R. E. Triemer, Evolution of the chloroplast genome in photosynthetic euglenoids: A comparison of *Eutreptia viridis* and *Euglena gracilis* (Euglenophyta). Protist 163, 832–843 (2012). 22364772 10.1016/j.protis.2012.01.002

[47] K. E. Wiegert, M. S. Bennett, R. E. Triemer, Tracing patterns of chloroplast evolution in euglenoids: Contributions from *Colacium vesiculosum* and *Strombomonas acuminata* (Euglenophyta). J. Eukaryot. Microbiol. 60, 214–221 (2013). 23351081 10.1111/jeu.12025

[48] T. Kleine, U. G. Maier, D. Leister, DNA transfer from organelles to the nucleus: The idiosyncratic genetics of endosymbiosis. Annu. Rev. Plant Biol. 60, 115–138 (2009). 19014347 10.1146/annurev.arplant.043008.092119

[49] U. S. Muchhal, S. D. Schwartzbach, Characterization of a *Euglena* gene encoding a polyprotein precursor to the light-harvesting chlorophyll *a*/*b*-binding protein of photosystem II. Plant Mol. Biol. 18, 287–299 (1992). 1731990 10.1007/BF00034956

[50] M. Kumazawa, H. Nishide, R. Nagao, N. Inoue-Kashino, J.-R. Shen, T. Nakano, I. Uchiyama, Y. Kashino, K. Ifuku, Molecular phylogeny of fucoxanthin-chlorophyll *a*/*c* proteins from *Chaetoceros gracilis* and Lhcq/Lhcf diversity. Physiol. Plant. 174, e13598 (2022). 34792189 10.1111/ppl.13598

[51] V. H. R. Schmid, H. Paulsen, J. Rupprecht, Identification of N- and C-terminal amino acids of Lhca1 and Lhca4 required for formation of the heterodimeric peripheral photosystem I antenna LHCI-730. Biochemistry 41, 9126–9131 (2002). 12119027 10.1021/bi016042x

[52] A. N. Melkozernov, R. E. Blankenship, Structural modeling of the Lhca4 Subunit of LHCI-730 peripheral antenna in photosystem I based on similarity with LHCII. J. Biol. Chem. 278, 44542–44551 (2003). 12923171 10.1074/jbc.M306777200

[53] K. Kato, Y. Nakajima, J. Xing, M. Kumazawa, H. Ogawa, J.-R. Shen, K. Ifuku, R. Nagao, Structural basis for molecular assembly of fucoxanthin chlorophyll *a*/*c*-binding proteins in a diatom photosystem I supercomplex. eLife 13, RP99858 (2024). 39480899 10.7554/eLife.99858 PMC11527431

[54] D. Carbonera, A. Agostini, M. Bortolus, L. Dall'Osto, R. Bassi, Violaxanthin and zeaxanthin may replace lutein at the L1 site of LHCII, conserving the interactions with surrounding chlorophylls and the capability of triplet-triplet energy transfer. Int. J. Mol. Sci. 23, 4812 (2022). 35563202 10.3390/ijms23094812 PMC9105099

[55] M. Havaux, L. Dall'Osto, S. Cuiné, G. Giuliano, R. Bassi, The effect of zeaxanthin as the only xanthophyll on the structure and function of the photosynthetic apparatus in *Arabidopsis thaliana*. J. Biol. Chem. 279, 13878–13888 (2004). 14722117 10.1074/jbc.M311154200

[56] F. G. Plumley, G. W. Schmidt, Reconstitution of chlorophyll *a*/*b* light-harvesting complexes: Xanthophyll-dependent assembly and energy transfer. Proc. Natl. Acad. Sci. U.S.A. 84, 146–150 (1987). 16593794 10.1073/pnas.84.1.146 PMC304159

[57] A. Natali, L. M. Roy, R. Croce, In vitro reconstitution of light-harvesting complexes of plants and green algae. J. Vis. Exp. 92, e51852 (2014). 10.3791/51852 PMC4692416 25350712

[58] S. Tamaki, K. Ozasa, T. Nomura, M. Ishikawa, K. Yamada, K. Suzuki, K. Mochida, Zeaxanthin is required for eyespot formation and phototaxis in *Euglena gracilis*. Plant Physiol. 191, 2414–2426 (2023). 36611254 10.1093/plphys/kiad001 PMC10069888

[59] L. Teng, X. Fan, D. R. Nelson, W. Han, X. Zhang, D. Xu, H. Renault, G. V. Markov, N. Ye, Diversity and evolution of cytochromes P450 in stramenopiles. Planta 249, 647–661 (2019). 30341489 10.1007/s00425-018-3028-1

[60] H. Cui, H. Ma, Y. Cui, X. Zhu, S. Qin, R. Li, Cloning, identification and functional characterization of two cytochrome P450 carotenoids hydroxylases from the diatom *Phaeodactylum tricornutum*. J. Biosci. Bioeng. 128, 755–765 (2019). 31277909 10.1016/j.jbiosc.2019.06.008

[61] S. Q. Zheng, E. Palovcak, J.-P. Armache, K. A. Verba, Y. Cheng, D. A. Agard, MotionCor2: Anisotropic correction of beam-induced motion for improved cryo-electron microscopy. Nat. Methods 14, 331–332 (2017). 28250466 10.1038/nmeth.4193 PMC5494038

[62] J. A. Mindell, N. Grigorieff, Accurate determination of local defocus and specimen tilt in electron microscopy. J. Struct. Biol. 142, 334–347 (2003). 12781660 10.1016/s1047-8477(03)00069-8

[63] D. Kimanius, L. Dong, G. Sharov, T. Nakane, S. H. W. Scheres, New tools for automated cryo-EM single-particle analysis in RELION-4.0. Biochem. J. 478, 4169–4185 (2021). 34783343 10.1042/BCJ20210708 PMC8786306

[64] N. Grigorieff, S. C. Harrison, Near-atomic resolution reconstructions of icosahedral viruses from electron cryo-microscopy. Curr. Opin. Struc. Biol. 21, 265–273 (2011). 10.1016/j.sbi.2011.01.008 PMC3088881 21333526

[65] T. Bepler, K. Kelley, A. J. Noble, B. Berger, Topaz-Denoise: General deep denoising models for cryoEM and cryoET. Nat. Commun. 11, 5208 (2020). 33060581 10.1038/s41467-020-18952-1 PMC7567117

[66] P. Emsley, B. Lohkamp, W. G. Scott, K. Cowtan, Features and development of *Coot*. Acta Crystallogr. D Biol. Crystallogr. 66, 486–501 (2010). 20383002 10.1107/S0907444910007493 PMC2852313

[67] P. D. Adams, P. V. Afonine, G. Bunkóczi, V. B. Chen, I. W. Davis, N. Echols, J. J. Headd, L.-W. Hung, G. J. Kapral, R. W. Grosse-Kunstleve, A. J. McCoy, N. W. Moriarty, R. Oeffner, R. J. Read, D. C. Richardson, J. S. Richardson, T. C. Terwilliger, P. H. Zwart, PHENIX: A comprehensive Python-based system for macromolecular structure solution. Acta Crystallogr. D Biol. Crystallogr. 66, 213–221 (2010). 20124702 10.1107/S0907444909052925 PMC2815670

[68] K. Yamashita, C. M. Palmer, T. Burnley, G. N. Murshudov, Cryo-EM single-particle structure refinement and map calculation using Servalcat. Acta Crystallogr. D Struct. Biol. 77, 1282–1291 (2021). 34605431 10.1107/S2059798321009475 PMC8489229

[69] V. B. Chen, W. B. Arendall III, J. J. Headd, D. A. Keedy, R. M. Immormino, G. J. Kapral, L. W. Murray, J. S. Richardson, D. C. Richardson, MolProbity: All-atom structure validation for macromolecular crystallography. Acta Crystallogr. D Biol. Crystallogr. 66, 12–21 (2010). 20057044 10.1107/S0907444909042073 PMC2803126

[70] B. A. Barad, N. Echols, R. Y.-R. Wang, Y. Cheng, F. DiMaio, P. D. Adams, J. S. Fraser, EMRinger: Side chain-directed model and map validation for 3D cryo-electron microscopy. Nat. Methods 12, 943–946 (2015). 26280328 10.1038/nmeth.3541 PMC4589481

[71] G. Pintilie, K. Zhang, Z. Su, S. Li, M. F. Schmid, W. Chiu, Measurement of atom resolvability in cryo-EM maps with *Q*-scores. Nat. Methods 17, 328–334 (2020). 32042190 10.1038/s41592-020-0731-1 PMC7446556

[72] L. L. C. Schrödinger, “The PyMOL molecular graphics system. Version 2.5.0.” (2021); www.pymol.org/.

[73] E. F. Pettersen, T. D. Goddard, C. C. Huang, G. S. Couch, D. M. Greenblatt, E. C. Meng, T. E. Ferrin, UCSF chimera—A visualization system for exploratory research and analysis. J. Comput. Chem. 25, 1605–1612 (2004). 15264254 10.1002/jcc.20084

[74] E. F. Pettersen, T. D. Goddard, C. C. Huang, E. C. Meng, G. S. Couch, T. I. Croll, J. H. Morris, T. E. Ferrin, UCSF ChimeraX: Structure visualization for researchers, educators, and developers. Protein Sci. 30, 70–82 (2021). 32881101 10.1002/pro.3943 PMC7737788

[75] P. Lamesch, T. Z. Berardini, D. Li, D. Swarbreck, C. Wilks, R. Sasidharan, R. Muller, K. Dreher, D. L. Alexander, M. Garcia-Hernandez, A. S. Karthikeyan, C. H. Lee, W. D. Nelson, L. Ploetz, S. Singh, A. Wensel, E. Huala, The Arabidopsis Information Resource (TAIR): Improved gene annotation and new tools. Nucleic Acids Res. 40, D1202–D1210 (2012). 22140109 10.1093/nar/gkr1090 PMC3245047

[76] D. Lang, K. K. Ullrich, F. Murat, J. Fuchs, J. Jenkins, F. B. Haas, M. Piednoel, H. Gundlach, M. Van Bel, R. Meyberg, C. Vives, J. Morata, A. Symeonidi, M. Hiss, W. Muchero, Y. Kamisugi, O. Saleh, G. Blanc, E. L. Decker, N. van Gessel, J. Grimwood, R. D. Hayes, S. W. Graham, L. E. Gunter, S. F. McDaniel, S. N. W. Hoernstein, A. Larsson, F.-W. Li, P.-F. Perroud, J. Phillips, P. Ranjan, D. S. Rokshar, C. J. Rothfels, L. Schneider, S. Shu, D. W. Stevenson, F. Thümmler, M. Tillich, J. C. Villarreal Aguilar, T. Widiez, G. K.-S. Wong, A. Wymore, Y. Zhang, A. D. Zimmer, R. S. Quatrano, K. F. X. Mayer, D. Goodstein, J. M. Casacuberta, K. Vandepoele, R. Reski, A. C. Cuming, G. A. Tuskan, F. Maumus, J. Salse, J. Schmutz, S. A. Rensing, The *Physcomitrella patens* chromosome-scale assembly reveals moss genome structure and evolution. Plant J. 93, 515–533 (2018). 29237241 10.1111/tpj.13801

[77] S. S. Merchant, S. E. Prochnik, O. Vallon, E. H. Harris, S. J. Karpowicz, G. B. Witman, A. Terry, A. Salamov, L. K. Fritz-Laylin, L. Maréchal-Drouard, W. F. Marshall, L.-H. Qu, D. R. Nelson, A. A. Sanderfoot, M. H. Spalding, V. V. Kapitonov, Q. Ren, P. Ferris, E. Lindquist, H. Shapiro, S. M. Lucas, J. Grimwood, J. Schmutz, P. Cardol, H. Cerutti, G. Chanfreau, C.-L. Chen, V. Cognat, M. T. Croft, R. Dent, S. Dutcher, E. Fernández, H. Fukuzawa, D. González-Ballester, D. González-Halphen, A. Hallmann, M. Hanikenne, M. Hippler, W. Inwood, K. Jabbari, M. Kalanon, R. Kuras, P. A. Lefebvre, S. D. Lemaire, A. V. Lobanov, M. Lohr, A. Manuell, I. Meier, L. Mets, M. Mittag, T. Mittelmeier, J. V. Moroney, J. Moseley, C. Napoli, A. M. Nedelcu, K. Niyogi, S. V. Novoselov, I. T. Paulsen, G. Pazour, S. Purton, J.-P. Ral, D. M. Riaño-Pachón, W. Riekhof, L. Rymarquis, M. Schroda, D. Stern, J. Umen, R. Willows, N. Wilson, S. L. Zimmer, J. Allmer, J. Balk, K. Bisova, C.-J. Chen, M. Elias, K. Gendler, C. Hauser, M. R. Lamb, H. Ledford, J. C. Long, J. Minagawa, M. D. Page, J. Pan, W. Pootakham, S. Roje, A. Rose, E. Stahlberg, A. M. Terauchi, P. Yang, S. Ball, C. Bowler, C. L. Dieckmann, V. N. Gladyshev, P. Green, R. Jorgensen, S. Mayfield, B. Mueller-Roeber, S. Rajamani, R. T. Sayre, P. Brokstein, I. Dubchak, D. Goodstein, L. Hornick, Y. W. Huang, J. Jhaveri, Y. Luo, D. Martínez, W. C. A. Ngau, B. Otillar, A. Poliakov, A. Porter, L. Szajkowski, G. Werner, K. Zhou, I. V. Grigoriev, D. S. Rokhsar, A. R. Grossman, The *Chlamydomonas* genome reveals the evolution of key animal and plant functions. Science 318, 245–250 (2007). 17932292 10.1126/science.1143609 PMC2875087

[78] R. Blanc-Mathieu, B. Verhelst, E. Derelle, S. Rombauts, F.-Y. Bouget, I. Carré, A. Château, A. Eyre-Walker, N. Grimsley, H. Moreau, B. Piégu, E. Rivals, W. Schackwitz, Y. Van de Peer, G. Piganeau, An improved genome of the model marine alga *Ostreococcus tauri* unfolds by assessing Illumina de novo assemblies. BMC Genomics 15, 1103 (2014). 25494611 10.1186/1471-2164-15-1103 PMC4378021

[79] J. E. W. Polle, K. Barry, J. Cushman, J. Schmutz, D. Tran, L. T. Hathwaik, W. C. Yim, J. Jenkins, Z. McKie-Krisberg, S. Prochnik, E. Lindquist, R. B. Dockter, C. Adam, H. Molina, J. Bunkenborg, E. Jin, M. Buchheim, J. Magnuson, Draft nuclear genome sequence of the halophilic and beta-carotene-accumulating green alga *Dunaliella salina* strain CCAP19/18. Genome Announc. 5, e01105-17 (2017). 29074648 10.1128/genomeA.01105-17 PMC5658486

[80] J. E. W. Polle, S. Calhoun, Z. McKie-Krisberg, S. Prochnik, P. Neofotis, W. C. Yim, L. T. Hathwaik, J. Jenkins, H. Molina, J. Bunkenborg, I. V. Grigoriev, K. Barry, J. Schmutz, E. Jin, J. C. Cushman, J. K. Magnuson, Genomic adaptations of the green alga *Dunaliella salina* to life under high salinity. Algal Res. 50, 101990 (2020).

[81] D. Bhattacharya, D. C. Price, C. X. Chan, H. Qiu, N. Rose, S. Ball, A. P. M. Weber, M. C. Arias, B. Henrissat, P. M. Coutinho, A. Krishnan, S. Zäuner, S. Morath, F. Hilliou, A. Egizi, M.-M. Perrineau, H. S. Yoon, Genome of the red alga *Porphyridium purpureum*. Nat. Commun. 4, 1941 (2013). 23770768 10.1038/ncomms2931 PMC3709513

[82] J. Kreplak, M.-A. Madoui, P. Cápal, P. Novák, K. Labadie, G. Aubert, P. E. Bayer, K. K. Gali, R. A. Syme, D. Main, A. Klein, A. Bérard, I. Vrbová, C. Fournier, L. d'Agata, C. Belser, W. Berrabah, H. Toegelová, Z. Milec, J. Vrána, H. Lee, A. Kougbeadjo, M. Térézol, C. Huneau, C. J. Turo, N. Mohellibi, P. Neumann, M. Falque, K. Gallardo, R. McGee, B. Tar'an, A. Bendahmane, J.-M. Aury, J. Batley, M.-C. Le Paslier, N. Ellis, T. D. Warkentin, C. J. Coyne, J. Salse, D. Edwards, J. Lichtenzveig, J. Macas, J. Doležel, P. Wincker, J. Burstin, A reference genome for pea provides insight into legume genome evolution. Nat. Genet. 51, 1411–1422 (2019). 31477930 10.1038/s41588-019-0480-1

[83] T. Yang, R. Liu, Y. Luo, S. Hu, D. Wang, C. Wang, M. K. Pandey, S. Ge, Q. Xu, N. Li, G. Li, Y. Huang, R. K. Saxena, Y. Ji, M. Li, X. Yan, Y. He, Y. Liu, X. Wang, C. Xiang, R. K. Varshney, H. Ding, S. Gao, X. Zong, Improved pea reference genome and pan-genome highlight genomic features and evolutionary characteristics. Nat. Genet. 54, 1553–1563 (2022). 36138232 10.1038/s41588-022-01172-2 PMC9534762

[84] K. Katoh, J. Rozewicki, K. D. Yamada, MAFFT online service: Multiple sequence alignment, interactive sequence choice and visualization. Brief. Bioinform. 20, 1160–1166 (2019). 28968734 10.1093/bib/bbx108 PMC6781576

[85] J. L. Steenwyk, T. J. Buida III, Y. Li, X.-X. Shen, A. Rokas, ClipKIT: A multiple sequence alignment trimming software for accurate phylogenomic inference. PLoS Biol. 18, e3001007 (2020). 33264284 10.1371/journal.pbio.3001007 PMC7735675

[86] B. Q. Minh, H. A. Schmidt, O. Chernomor, D. Schrempf, M. D. Woodhams, A. von Haeseler, R. Lanfear, IQ-TREE 2: New models and efficient methods for phylogenetic inference in the genomic era. Mol. Biol. Evol. 37, 1530–1534 (2020). 32011700 10.1093/molbev/msaa015 PMC7182206

[87] G. Bianchini, P. Sánchez-Baracaldo, TreeViewer: Flexible, modular software to visualise and manipulate phylogenetic trees. Ecol. Evol. 14, e10873 (2024). 38314311 10.1002/ece3.10873 PMC10834882

[88] Y. N. Yamamoto, T. Suzuki, Y. Ueno, T. Tomo, N. Dohmae, A. Takabayashi, R. Nagao, Biochemical and phylogenetic analyses of light-harvesting complexes from *Tetraselmis striata*. Photosynth. Res. 163, 32 (2025). 40418499 10.1007/s11120-025-01152-7

