# Supplementary material for: Structural insights into the divergent evolution of a photosystem I supercomplex in Euglena gracilis
Source: Sci Adv. 2025 Oct 31;11(44):eaea6241. doi: 10.1126/sciadv.aea6241 (PMC12577709; doi:10.1126/sciadv.aea6241)
Supplement: Supplementary file 1 — Figs. S1 to S9 Tables S1 to S9 [file sciadv.aea6241_sm.pdf]

Science Advances
AAAS

Supplementary Materials for

Structural insights into the divergent evolution of a photosystem I supercomplex in *Euglena gracilis*

Koji Kato *et al.*

Corresponding author: Jian-Ren Shen, shen@cc.okayama-u.ac.jp;
Atsushi Takabayashi, takabayashi@lowtem.hokudai.ac.jp; Ryo Nagao, nagry@shizuoka.ac.jp

Sci. Adv. **11**, eaea6241 (2025)
DOI: 10.1126/sciadv.aea6241

This PDF file includes:

Figs. S1 to S9
Tables S1 to S9

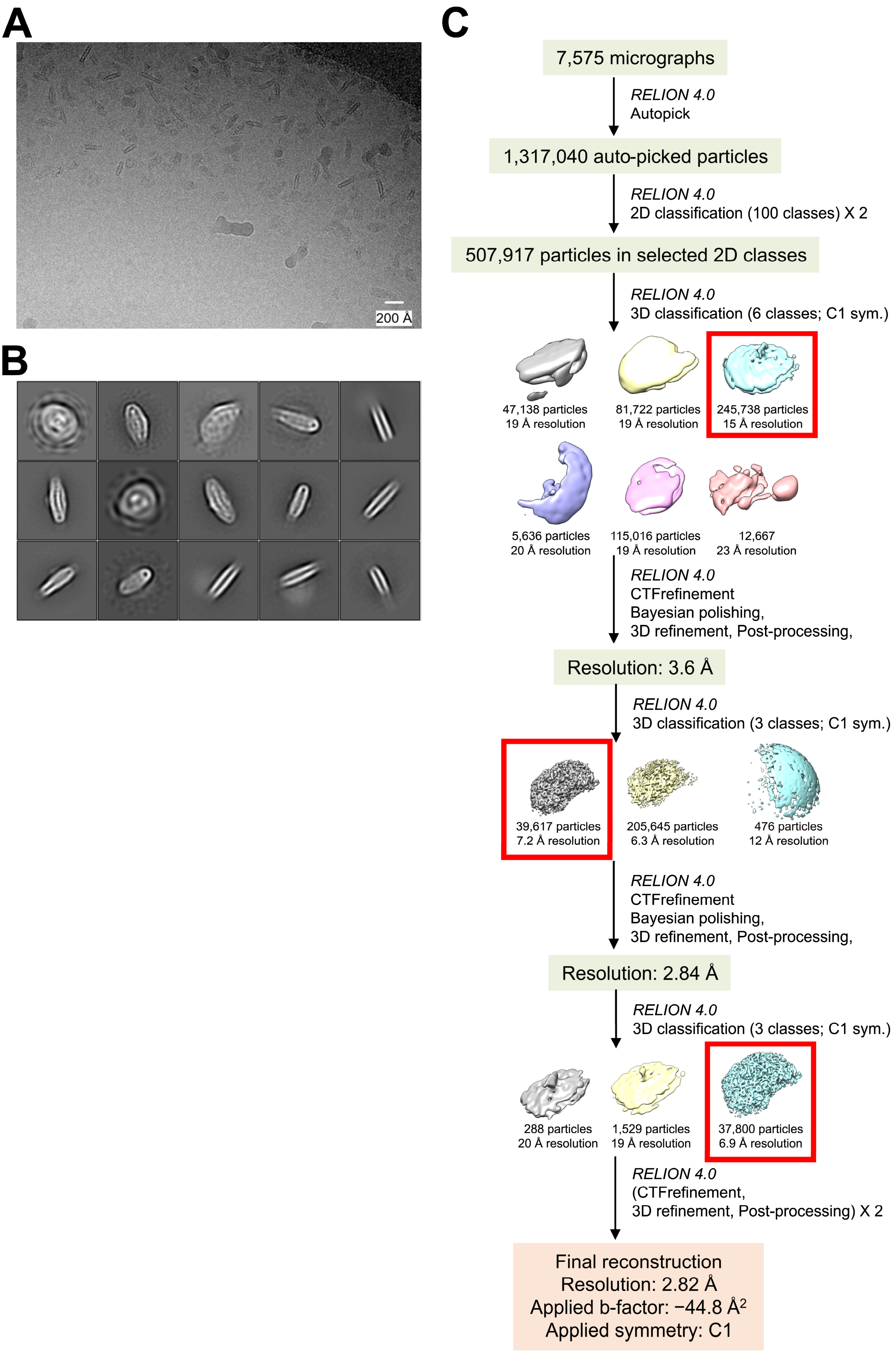

Fig. S1 | Cryo-EM data collection and processing of PSI-LHCI.

A, A representative cryo-EM micrograph of PSI-LHCI from 7,575 micrographs. **B,** Representative 2D classes of PSI-LHCI. The box size is 361 Å. **C,** A schematic flowchart showing

the classification scheme and data processing for PSI-LHCI. The overall PSI-LHCI structure was reconstructed at a resolution of 2.82 Å from 37,800 particles. See Methods section for more details.

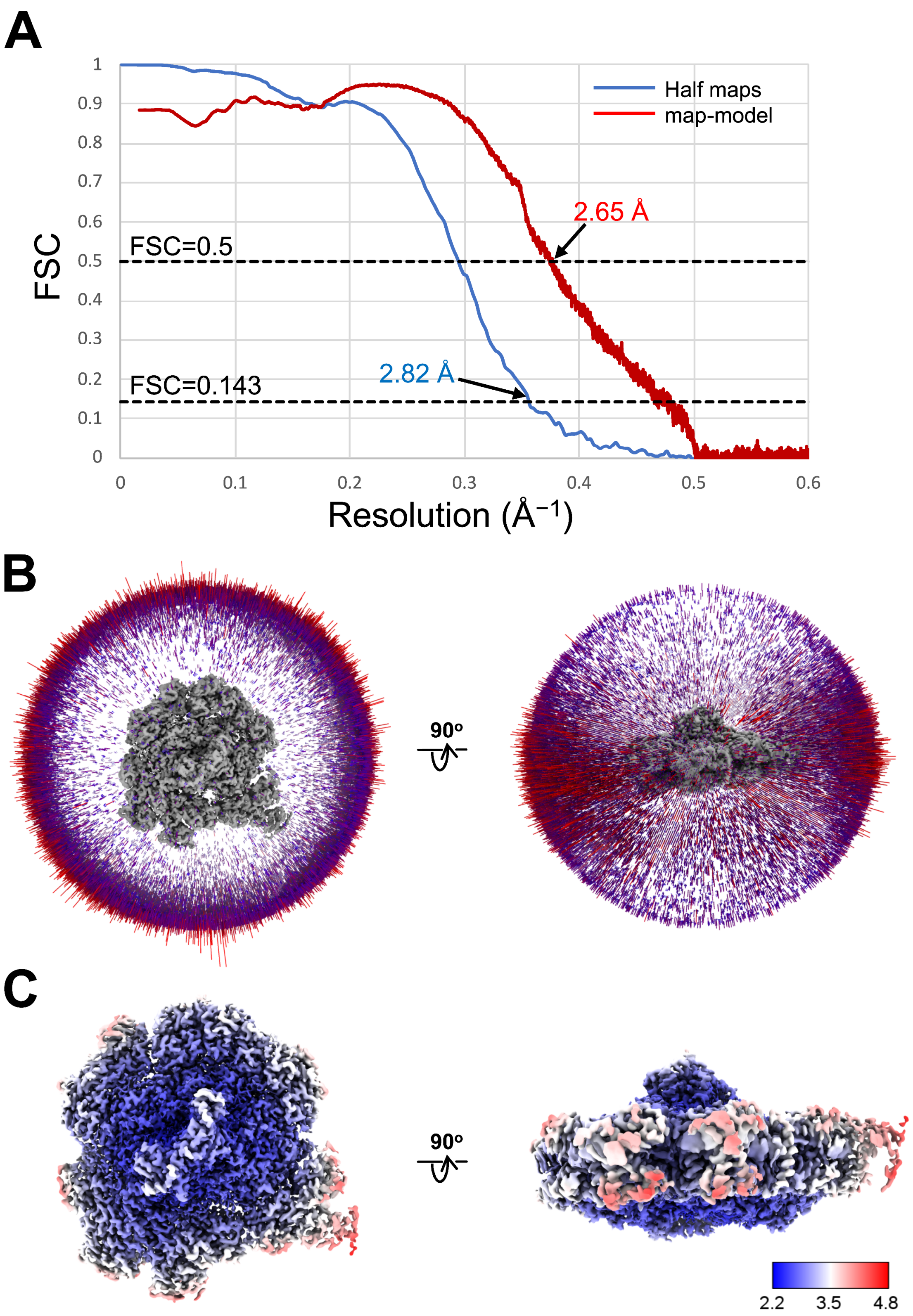

Fig. S2 | Evaluation of the cryo-EM map quality.

A, FSC curves of PSI-LHCI for independently refined half maps (blue) and map-minus-model (red). **B,** Angular distributions of the particles used for the reconstruction of PSI-LHCI. Each cylinder represents one view, and the height of the cylinder is proportional to the number of particles for that view. **C,** Local resolution maps of PSI-LHCI.

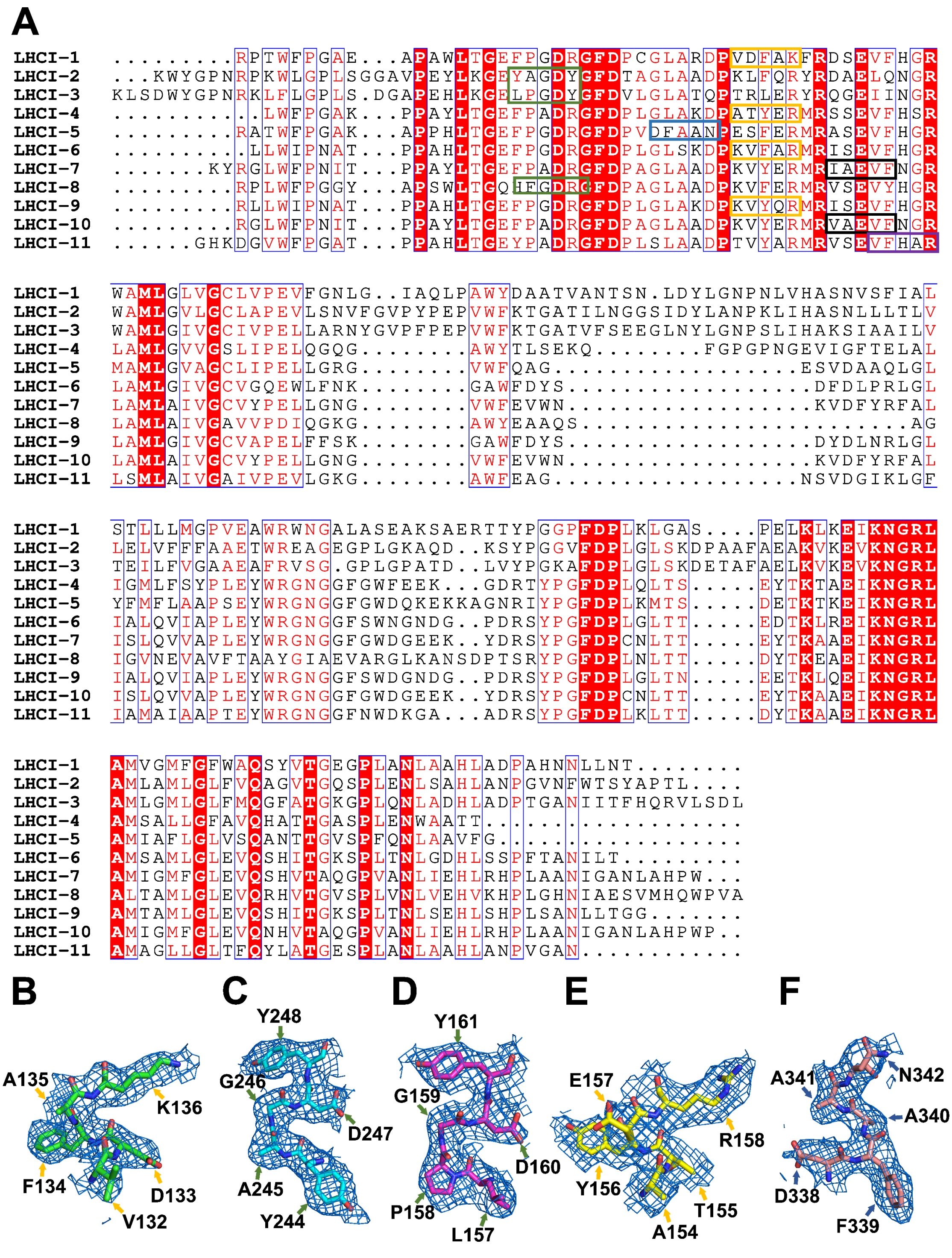

A
LHCI-1 ....RPTWFPGAE...APAWLTGEFPGDRGFDPCGLARDPVDFAKFRDSEVFHGR
LHCI-2 ...KWYGPNRPKWLGPLSGGAVPEYLKGEYAGDYGFDTAGLAADPKLFQRYRDAELQNGR
LHCI-3 KLSDWYGPNRKLFLGPLS.DGAPEHLKGELPGDYGFDVLGLATQPTRLERYRQGEIINGR
LHCI-4 ....LWFPGAK...PPAYLTGEFPADRGFDPLGLAKDPATYERMRSSEVFHSR
LHCI-5 ....RATWFPGAK...APPHLTGEFPGDRGFDPVDFAANPESFERMRASEVFHGR
LHCI-6 ....LLWIPNAT...PPAHLTGEFPGDRGFDPLGLSKDPKVFARMRISEVFHGR
LHCI-7 ....KYRGLWFPNIT...PPAYLTGEFPADRGFDPAGLAADPKVYERMRIAEVFNGR
LHCI-8 ....RPLWFPGGY...APSWLTGQHFGDRGFDPAGLAADPKVFERMRVSEVYHGR
LHCI-9 ....RLLWIPNAT...PPAHLTGEFPGDRGFDPLGLAKDPKVYQRMRISEVFHGR
LHCI-10 ....RGLWFPNIT...PPAYLTGEFPADRGFDPAGLAADPKVYERMRVAEVFNGR
LHCI-11 .....GHKDGVWFPGAT...PPAHLTGEYPADRGFDPLSLAADPTVYARMRVSEVFHAR
LHCI-1 WAMLGLVGCLVPEVFGNLG..IAQLPAWYDAATVANTSN.LDYLGNPNLVHASNVSFIAL
LHCI-2 WAMLGVLGCLAPEVLSNVFGVPYPEPVWFKTGATILNGGSIDYLANPKLIHASNLLLTLV
LHCI-3 WAMLGIVGCIVPELLARNYGVPFPEPVWFKTGATVFSEEGLNYLGNPSLIHAKSIAAILV
LHCI-4 LAMLGVVGSLIPELQGQG........AWYTLSEKQ........FGPGPNGEVIGFTELAL
LHCI-5 MAMLGVAGCLIPELLGRG........VWFQAG.................ESVDAAQLGL
LHCI-6 LAMLGIVGCVGQEWLFNK........GAWFDYS.................DFDLPRLGL
LHCI-7 LAMLAIVGCVYPELLGNG........VWFEVWN.................KVDFYRFAL
LHCI-8 LAMLAIVGAVVPDIQGKG........AWYEAAQS.......................AG
LHCI-9 LAMLGIVGCVAPELFFSK........GAWFDYS.................DYDLNRLGL
LHCI-10 LAMLAIVGCVYPELLGNG........VWFEVWN.................KVDFYRFAL
LHCI-11 LSMLAIVGAIVPEVLGKG........AWFEAG.................NSVDGIKLGF
LHCI-1 STLLLMGPVEAWRWNGALASEAKSAERTTYPGGPFDPLKLGAS....PELKLKEIKNGRL
LHCI-2 LELVFFFAAETWREAGEGPLGKAQD..KSYPGGVFDPLGLSKDPAAFAEAKVKEVKNGRL
LHCI-3 TEILFVGAAEAFRVSG.GPLGPATD..LVYPGKAFDPLGLSKDETAFAELKVKEVKNGRL
LHCI-4 IGMLFSYPLEYWRGNGGFGWFEEK...GDRTYPGFDPLQLTS.....EYTKTAEIKNGRL
LHCI-5 YFMFLAAPSEYWRGNGGFGWDQKEKKAGNRIYPGFDPLKMTS.....DETKTKEIKNGRL
LHCI-6 IALQVIAPLEYWRGNGGFSWNGNDG..PDRSYPGFDPLGLTT.....EDTKLREIKNGRL
LHCI-7 ISLQVVAPLEYWRGNGGFGWDGEEK..YDRSYPGFDPCNLTT.....EYTKAAEIKNGRL
LHCI-8 IGVNEVAVFTAAYGIAEVARGLKANSDPTSRYPGFDPLNLTT.....DYTKEAEIKNGRL
LHCI-9 IALQVIAPLEYWRGNGGFSWDGNDG..PDRSYPGFDPLGLTN.....EETKLQEIKNGRL
LHCI-10 ISLQVVAPLEYWRGNGGFGWDGEEK..YDRSYPGFDPCNLTT.....EYTKAAEIKNGRL
LHCI-11 IAMAIAAPTEYWRGNGGFNWDKGA...ADRSYPGFDPLKLTT.....DYTKAAEIKNGRL
LHCI-1 AMVGMFGFWAQSYVTGEGPLANLAAHLADPAHNNLLNT........
LHCI-2 AMLAMLGLFVQAGVTGQSPLENLSAHLANPGVNFWTSYAPTL....
LHCI-3 AMLGMLGLFMQGFATGKGPLQNLADHLADPTGANIITFHQRVLSDL
LHCI-4 AMSALLGFAVQHATTGASPLENWAATT...................
LHCI-5 AMIAFLGLVSQANTTGVSPFQNLAAVFG..................
LHCI-6 AMSAMLGLEVQSHITGKSPLTNLGDHLSSPFTANILT.........
LHCI-7 AMIGMFGLEVQSHVTAQGPVANLIEHLRHPLAANIGANLAHPW...
LHCI-8 ALTAMLGLEVQRHVTGVSPLVNLVEHVKHPLGHNIAESVMHQWPVA
LHCI-9 AMTAMLGLEVQSHITGKSPLTNLSEHLSHPLSANLLTGG.......
LHCI-10 AMIGMFGLEVQNHVTAQGPVANLIEHLRHPLAANIGANLAHPWP..
LHCI-11 AMAGLLGLTFQYLATGESPLANLAAHLANPVGAN............
B
A135
K136
F134
D133
V132
C
Y248
G246
D247
A245
Y244
D
Y161
G159
D160
P158
L157
E
E157
R158
Y156
T155
A154
F
A341
N342
A340
D338
F339

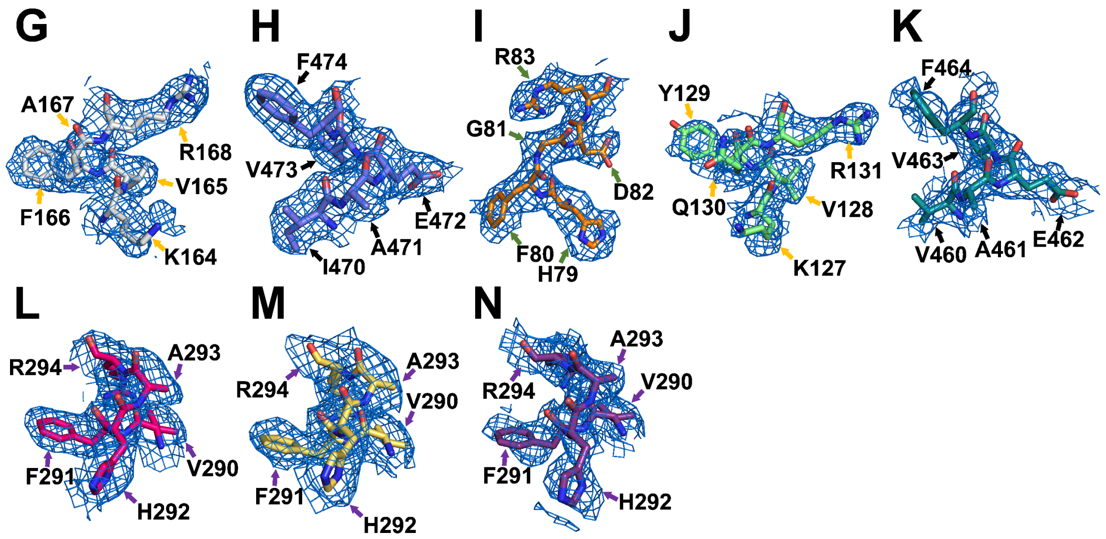

Fig. S3 | Characteristic amino acid residues used for the identification of each LHCI subunit. **A,** Multiple sequence alignment of the LHCI proteins of *E. gracilis* strain Z using ClustalW (https://www.genome.jp/tools-bin/clustalw) and ESPript (https://espript.ibcp.fr/ESPript/cgi-bin/ESPript.cgi). Unique residues are enclosed in boxes with different colors, which were used for the identification of the LHCI subunits. Residue numbering was omitted from the alignment because reliable sequence assignment was limited to regions supported by both the density maps and transcriptome data. LHCI-12 and LHCI-13 share the same sequence as LHCI-11 within the structurally resolved regions (see text). **B–N,** Characteristic maps and amino acid residues of LHCI-1 (**B**), LHCI-2 (**C**), LHCI-3 (**D**), LHCI-4 (**E**), LHCI-5 (**F**), LHCI-6 (**G**), LHCI-7 (**H**), LHCI-8 (**I**), LHCI-9 (**J**), LHCI-10 (**K**), LHCI-11 (**L**), LHCI-12 (**M**), and LHCI-13 (**N**). The densities and models are shown as meshes and sticks, respectively. The characteristic amino acids are labeled with boxes of the same color as shown in panel **A**.

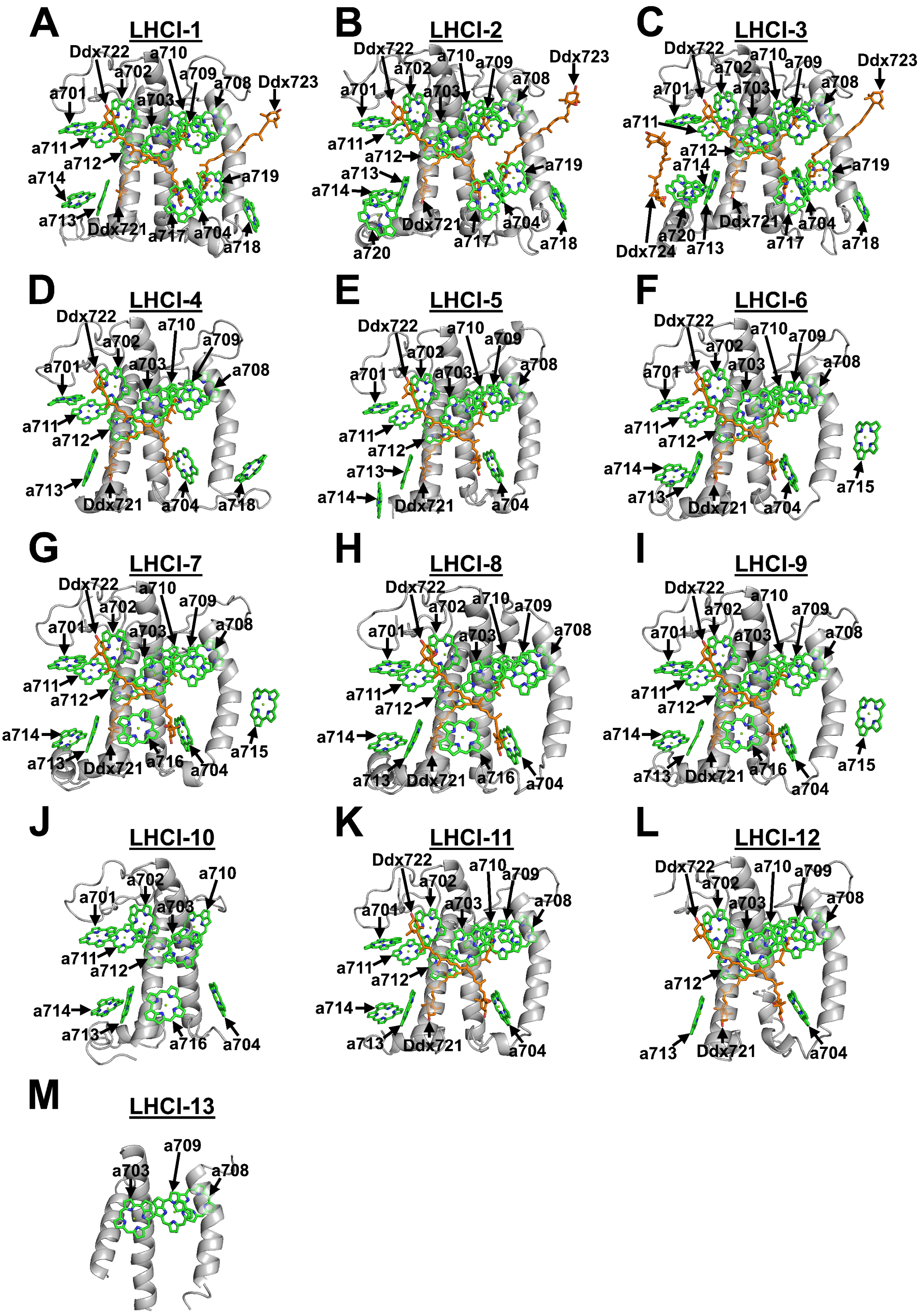

Fig. S4 | Structures of the *E. gracilis* LHCIs.

A–M, Structures of LHCI-1 to LHCI-13 depicted as transparent cartoons for proteins and sticks for Chls and Cars with different colors. Only rings of the Chl molecules are depicted. Green, Chl *a*; orange, Ddx.

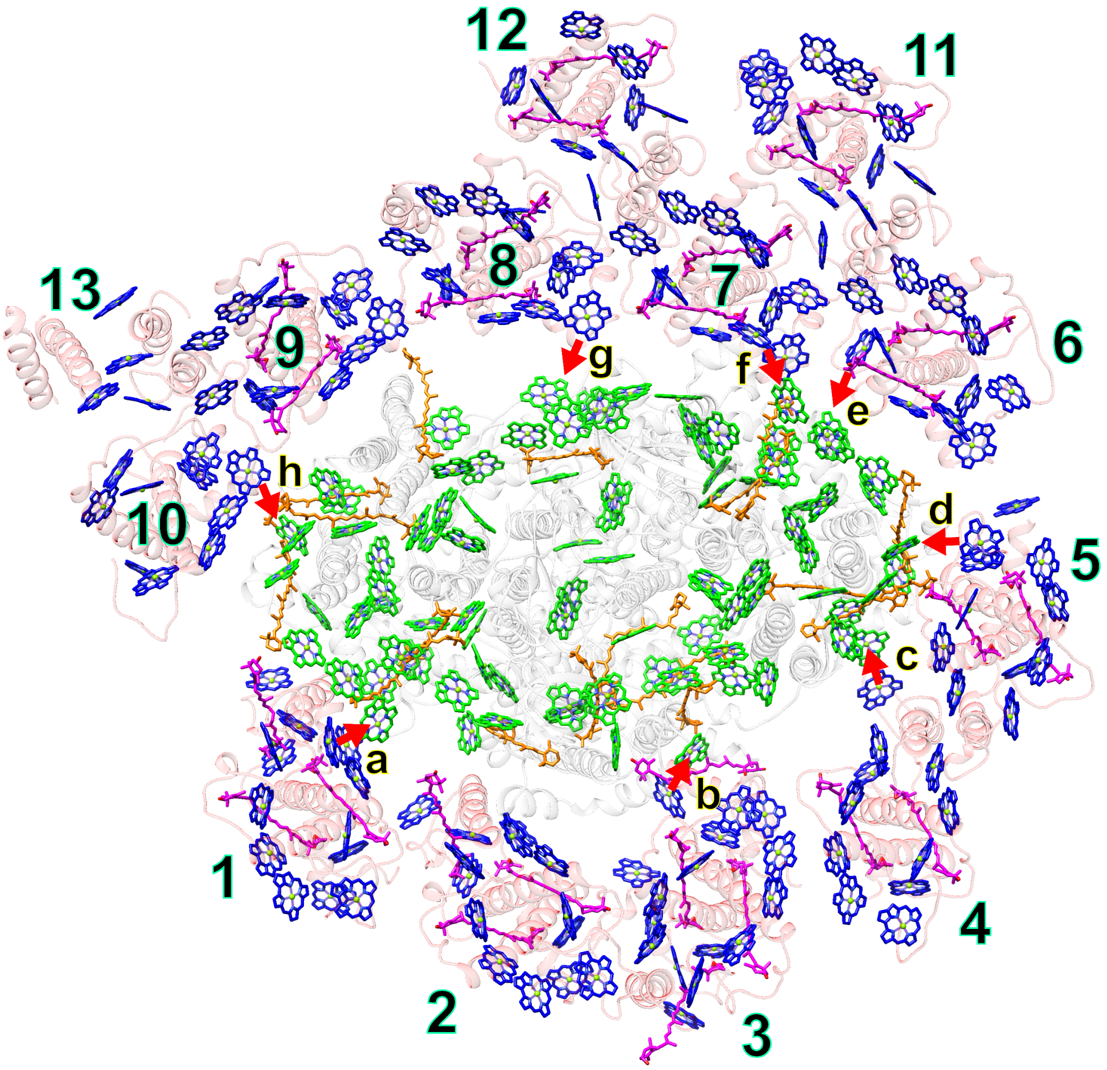

Fig. S5 | Arrangement of pigment molecules within the *E. gracilis* PSI-LHCI and possible excitation-energy-transfer pathways from LHCIs to PSI core.

The structure is viewed from the stromal side. The protein structures of PSI core and LHCIs are displayed in transparent cartoons and colored grey and red, respectively. The numbers 1–13 correspond to LHCI-1 to LHCI-13, respectively. Chls and Cars are shown as sticks, and only rings of the Chl molecules are depicted. Green, Chls *a* in PSI; orange, BCRs; blue, Chls *a* in LHCIs; magenta, Ddxs. Red arrows labeled with letters a–h indicate possible excitation-energy-transfer pathways based on close physical interactions among Chls (see Main text).

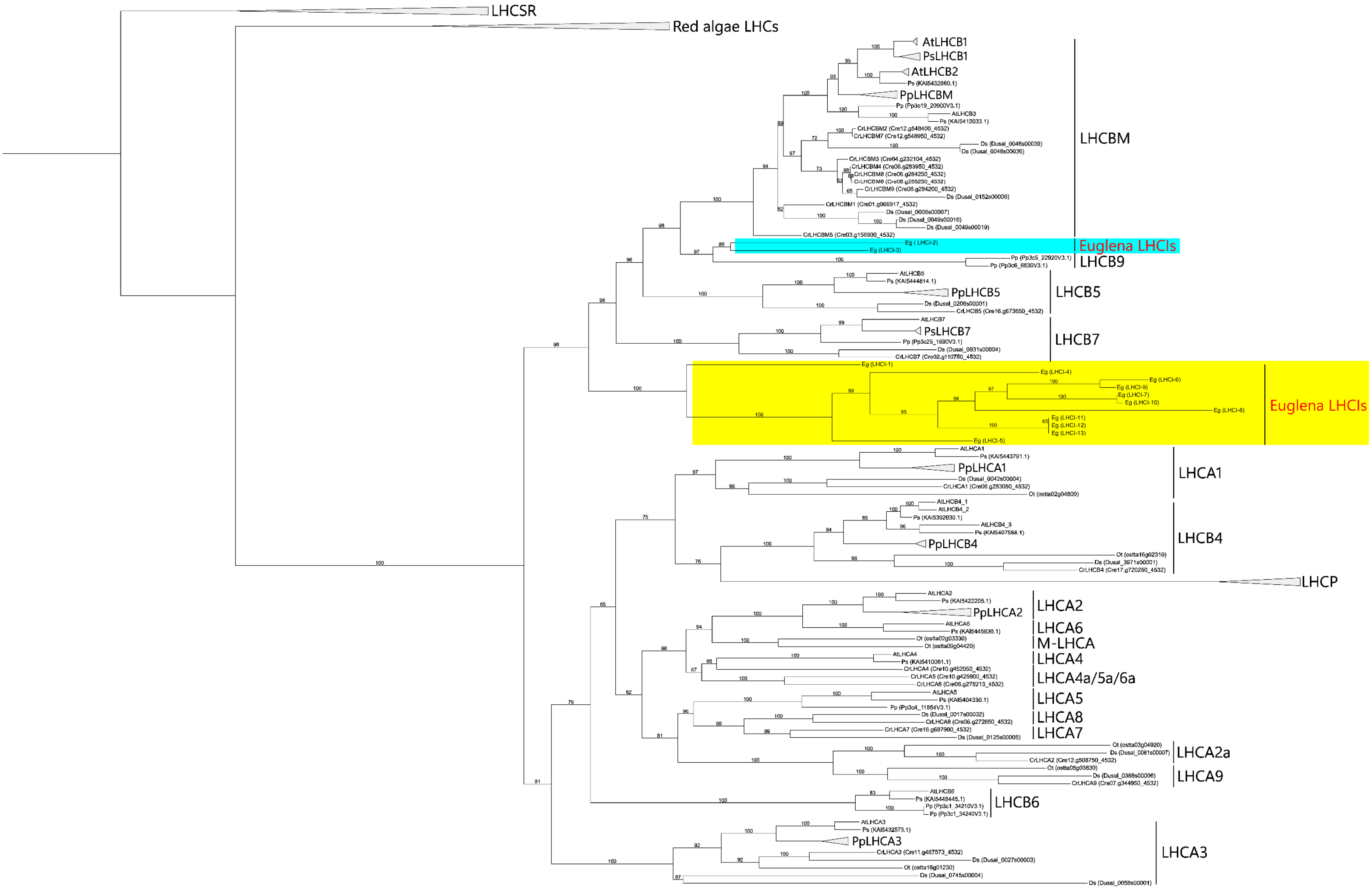

Fig. S6 | Collapsed ML tree of *Euglena* LHCI proteins.

An ML tree including the *E. gracilis* LHCI proteins (LHCI-1 to LHCI-13), which are highlighted in cyan and yellow, was constructed using IQ-TREE. Some LHC clades were collapsed using TreeViewer, and the uncollapsed tree is shown in fig. S7. Ultrafast bootstrap values (1,000 replicates) are indicated at the branches. LHC nomenclature follows our recent study (ref 88 in the main text). M-LHCA represents Mamiellales-specific LHCI. To avoid confusion arising from functional and evolutionary differences, we distinguish some green algae-specific LHCI proteins from their land plant counterparts with the same names by appending the subscript "a" (e.g., LHCA2a, LHCA4a, LHCA5a, and LHCA6a). At (*Arabidopsis thaliana*), Ps (*Pisum sativum*), Pp (*Physcomitrium patens*), Ot (*Ostreococcus tauri*), Cr (*Chlamydomonas reinhardtii*), Ds (*Dunaliella salina*), Eg (*Euglena gracilis*).

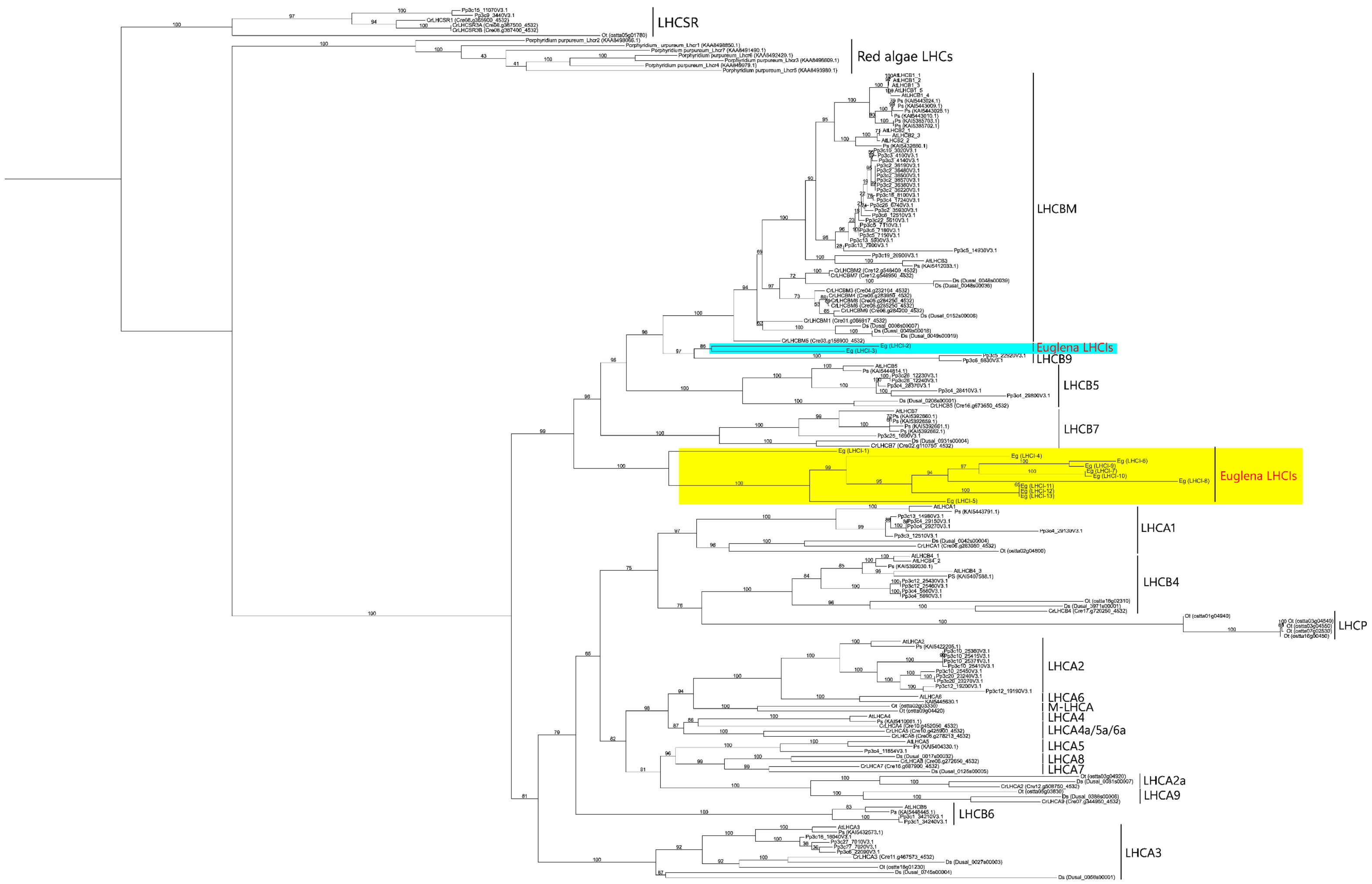

Fig. S7 | Uncollapsed ML tree of *E. gracilis* LHCI proteins.

An uncollapsed ML tree including *E. gracilis* LHCI proteins (LHCI-1 to LHCI-13), which are highlighted in cyan and yellow, was constructed using IQ-TREE. Ultrafast bootstrap values (1,000 replicates) are indicated at the branches. LHC nomenclature follows our recent study (ref 88 in the main text). M-LHCA represents Mamiellales-specific LHCI. To avoid confusion arising from functional and evolutionary differences, we distinguish some green algae-specific LHCI proteins from their land plant counterparts with the same names by appending the subscript "a" (e.g., LHCA2a, LHCA4a, LHCA5a, and LHCA6a). At (*Arabidopsis thaliana*), Ps (*Pisum sativum*), Pp (*Physcomitrium patens*), Ot (*Ostreococcus tauri*), Cr (*Chlamydomonas reinhardtii*), Ds (*Dunaliella salina*), Eg (*Euglena gracilis*).

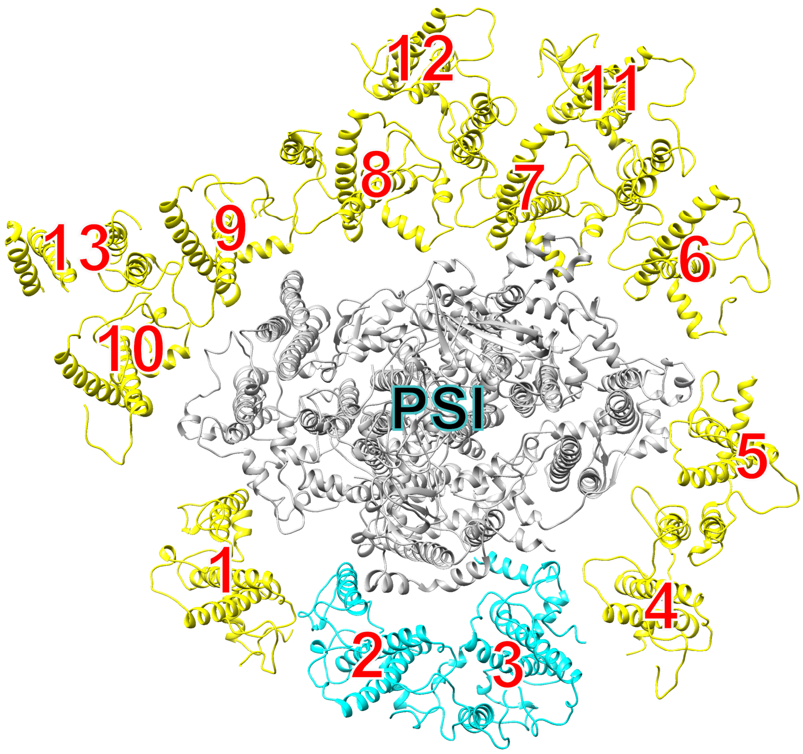

Fig. S8 | Structural distribution of LHCI subunits based on phylogenetic analysis.

The *Euglena* PSI-LHCI structure is viewed from the stromal side. The numbers 1–13 corresponds to LHCI-1 to LHCI-13, respectively. The LHCI subunits highlighted in cyan and yellow correspond to the respective clades indicated by the same colors in Fig. 5A.

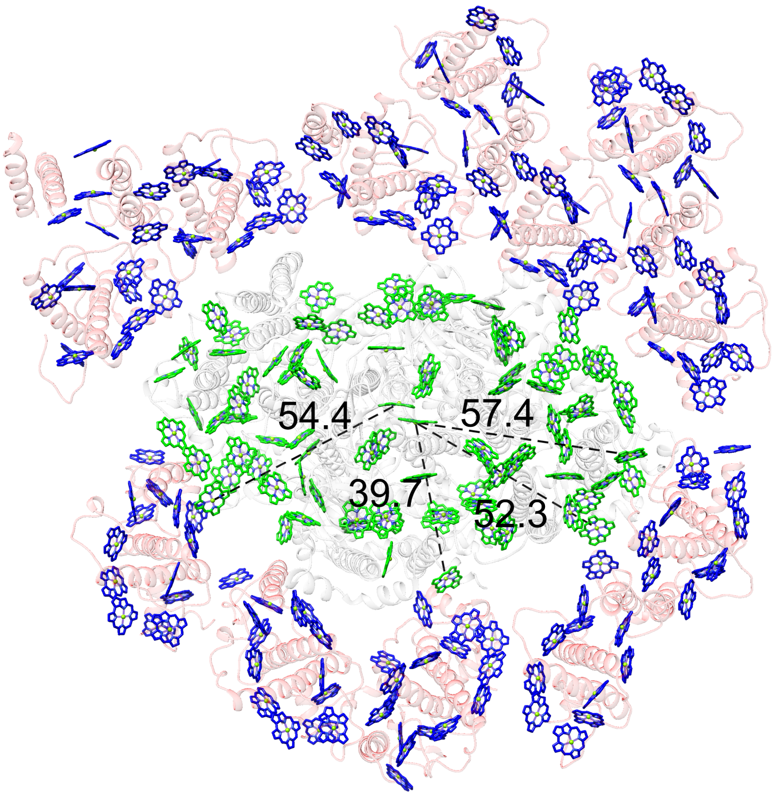

Fig. S9 | Distances from peripheral Chls to P700 within PSI.

The *Euglena* PSI-LHCI structure is viewed from the stromal side. Only rings of the Chl molecules are shown as sticks. Dashed lines indicate Chl-Chl distances, with values labeled in Å. Chls in PSI are colored green, and those in LHCIs are colored blue. Protein structures are shown as transparent cartoons.

Table S1 | Cryo-EM data collection and structural analysis statistics.

Complex	PSI-LHCI
PDB ID	9VFJ
EMDB ID	EMD-65026
Data collection and processing	
Magnification	60000
Voltage (kV)	300
Electron exposure (e^{-}/Å)	50
Defocus range (µm)	−1.8 to −1.2
Pixel size (Å)	0.752
Symmetry imposed	C1
Initial particle images (no.)	507,565
Final particle images (no.)	56,060
Map resolution (Å)	2.82
FSC threshold	0.143
Refinement	
Initial Model used	De novo model building
Model resolution (Å)	2.65
FSC threshold	0.5
Map sharpening B factor ($Å^2$)	−44.8
Model composition	
Non-hydrogen atoms	48,244
Protein residues	4,252
Ligand molecules	342
Water molecules	44
B factors ($Å^2$)	
Protein	86.1
Ligand	91.0
Water	46.5
R.m.s deviations	
Bond lengths (Å)	0.029
Bond angles (°)	3.08
Validation	
MolProbity score	2.25
Clashscore	7.54
Poor rotamers (%)	4.17
EMRinger score	3.98
Ramachandran plot	
Favored (%)	94.62
Allowed (%)	4.88
Disallowed (%)	0.50

Table S2 | Averaged *Q*-scores in each subunit.

Subunit	Averaged *Q*-score	
	Postprocessed map	Denoised map
PsaA	0.80	0.79
PsaB	0.80	0.79
PsaC	0.81	0.79
PsaD	0.73	0.75
PsaE	0.73	0.75
PsaF	0.76	0.77
PsaJ	0.78	0.78
PsaM	0.72	0.74
LHCI-1	0.68	0.72
LHCI-2	0.71	0.74
LHCI-3	0.68	0.72
LHCI-4	0.59	0.67
LHCI-5	0.70	0.73
LHCI-6	0.64	0.69
LHCI-7	0.74	0.76
LHCI-8	0.67	0.72
LHCI-9	0.62	0.69
LHCI-10	0.50	0.59
LHCI-11	0.59	0.67
LHCI-12	0.53	0.62
LHCI-13	0.46	0.58

Table S3 | Cofactors assigned in each subunit of the PSI-LHCI structure.

Protein	Chlorophyll	Carotenoid	Lipid	Other
PsaA	44 Chl *a*<br>1 Chl *a′*	6 BCR	2 LHG	1 [4Fe-4S] cluster,<br>1 phylloquinone
PsaB	41 Chl *a*	7 BCR	1 LHG<br>1 DGD	1 phylloquinone
PsaC	-	-	-	2 [4Fe-4S] cluster
PsaD	-	-	-	-
PsaE	-	-	-	-
PsaF	2 Chl *a*	1 BCR	-	-
PsaJ	1 Chl *a*	2 BCR	-	-
PsaM	-	1 BCR	-	-
LHCI-1	14 Chl *a*	3 Ddx	1 LHG	-
LHCI-2	15 Chl *a*	3 Ddx	1 LHG	-
LHCI-3	15 Chl *a*	4 Ddx	1 LHG	-
LHCI-4	11 Chl *a*	2 Ddx	1 LHG	-
LHCI-5	11 Chl *a*	2 Ddx	1 LHG	-
LHCI-6	12 Chl *a*	2 Ddx	1 LHG	-
LHCI-7	13 Chl *a*	2 Ddx	1 LHG	-
LHCI-8	12 Chl *a*	2 Ddx	1 LHG	-
LHCI-9	13 Chl *a*	2 Ddx	1 LHG	-
LHCI-10	10 Chl *a*	-	1 LHG	-
LHCI-11	11 Chl *a*	2 Ddx	1 LHG	-
LHCI-12	8 Chl *a*	2 Ddx	-	-
LHCI-13	3 Chl *a*	-	-	-
Total	237	43	15	5

BCR, *β*-carotene; Ddx, diadinoxanthin; Chl *a*, chlorophyll *a*; Chl *a′*, chlorophyll *a* epimer; DGD, digalactosyl diacyl glycerol; LHG, dipalmitoyl phosphatidyl glycerol.

Table S4 | LHCI proteins identified in the PSI-LHCI structure and their RMSD values with LHCI-4

Protein	RMSD (Å)/Aligned Cα atoms
LHCI-1	1.46/154
LHCI-2	1.38/151
LHCI-3	1.49/148
LHCI-4	0.00/169
LHCI-5	0.94/159
LHCI-6	0.98/157
LHCI-7	0.98/157
LHCI-8	1.11/147
LHCI-9	1.14/157
LHCI-10	0.80/122
LHCI-11	0.84/158
LHCI-12	0.87/150
LHCI-13	0.71/73

Table S5 | Chls and their ligands in each of the LHCI subunits.

Protein	Chlorophyll/ligand
LHCI-1	a701/W102, a702/E141, a703/H144, a704/w27[2], a708/w20[2], a709/E213, a710/E253, a711/LHG731, a712/N256, a713/Q270, a714/H285, a717/w21[2], a718/V193, a719/w19[2]
LHCI-2	a701/W227, a702/E269, a703/N272, a704/w23[2], a708/w24[2], a709/E344, a710/E386, a711/LHG731, a712/N389, a713/Q403, a714/H418, a717/w25[2], a718/I324, a719/w22[2], a720/-[1]
LHCI-3	a701/F141, a702/E182, a703/N185, a704/w26[2], a708/w28[2], a709/E257, a710/E298, a711/LHG731, a712/N301, a713/Q315, a714/H330, a717/w30[2], a718/I237, a719/w29[2], a720/H343
LHCI-4	a701/W124, a702/E163, a703/H166, a704/w43[2], a708/w31[2], a709/E222, a710/E258, a711/LHG731, a712/N261, a713/Q275, a718/w32[2]
LHCI-5	a701/W314, a702/E353, a703/H356, a704/w36[2], a708/w33[2], a709/E402, a710/E441, a711/LHG731, a712/N444, a713/Q458, a714/V473
LHCI-6	a701/W134, a702/E173, a703/H176, a704/Q190, a708/w34[2], a709/E222, a710/E259, a711/LHG731, a712/N262, a713/Q276, a714/H291, a715/Q216
LHCI-7	a701/W433, a702/E472, a703/N475, a704/w16[2], a708/w17[2], a709/E521, a710/E558, a711/LHG731, a712/N561, a713/Q575, a714/H590, a715/Q515, a716/E573
LHCI-8	a701/W65, a702/E104, a703/H107, a704/w18[2], a708/w35[2], a709/E154, a710/E186, a711/LHG731, a712/N189, a713/Q203, a714/H218, a716/E201
LHCI-9	a701/W97, a702/E136, a703/H139, a704/w41[2], a708/w42[2], a709/E185, a710/E222, a711/LHG731, a712/N225, a713/Q239, a714/H254, a715/Q179, a716/E237
LHCI-10	a701/W423, a702/E462, a703/N465, a704/-[1], a710/E548, a711/LHG731, a712/N551, a713/Q565, a714/H580, a716/E563
LHCI-11	a701/W250, a702/E289, a703/H292, a704/w44[2], a708/-[1], a709/E338, a710/E374, a711/LHG731, a712/N377, a713/Q391, a714/H406
LHCI-12	a702/E289, a703/H292, a704/-[1], a708/-[1], a709/E338, a710/E374, a712/N377, a713/Q391
LHCI-13	a703/H292, a708/-[1], a709/E338

[1]The ligands of Chls may be water or lipid molecules which cannot be identified due to weak densities.

[2]Water molecules.

Table S6 | Correspondence between the numbering of the pigment molecule in the PsaA subunit used in the text and that in the PDB file.

	**PsaA**
**Chl in the text**	
862	848 (A)

Table S7 | Correspondence between the numbering of pigments in each LHCI subunit used in the text and those in the PDB file.

	LHCI-1	LHCI-2	LHCI-3	LHCI-4	LHCI-5	LHCI-6	LHCI-7
**Chls in the text**	**PDB No. (Chain ID)**	**PDB No. (Chain ID)**	**PDB No. (Chain ID)**	**PDB No. (Chain ID)**	**PDB No. (Chain ID)**	**PDB No. (Chain ID)**	**PDB No. (Chain ID)**
701	504 (1)	503 (2)				903 (6)	702 (7)
702	505(1)	504 (2)				904 (6)	703 (7)
703	506(1)	505 (2)				905 (6)	704 (7)
704	507(1)	506 (2)				906 (6)	705 (7)
708	508(1)	507 (2)	705 (3)	705 (4)	705 (5)	907 (6)	706 (7)
709	509(1)	508(2)	706 (3)	706 (4)	706 (5)	908 (6)	707 (7)
710	510 (1)	509(2)	707 (3)	707 (4)	707 (5)	909 (6)	708 (7)
711	511 (1)	510 (2)	708 (3)	708 (4)	708 (5)	910 (6)	709 (7)
712	512 (1)	511 (2)	709 (3)	709 (4)	709 (5)	911 (6)	710 (7)
713	513 (1)	512 (2)	710 (3)	710 (4)	710 (5)	912 (6)	711 (7)
714	514 (1)	513 (2)	711 (3)		711 (5)	913 (6)	712 (7)
715						914 (6)	713 (7)
716							714 (7)
717	515 (1)	514 (2)	712 (3)				
718	516 (1)	515 (2)	713 (3)	711 (4)			
719	517 (1)	516 (2)	714 (3)				
720		517 (2)	715 (3)				
**Cars in the text**							
721	518 (1)	518 (2)	716 (3)	712 (4)	712 (5)	915 (6)	715 (7)
722	519 (1)	519 (2)	717 (3)	713 (4)	713 (5)	916 (6)	716 (7)
723	520 (1)	520 (2)	718 (3)				
724			104 (J)*				

*Chain in the adjacent unit.

	LHCI-8	LHCI-9	LHCI-10	LHCI-11	LHCI-12	LHCI-13
**Chls in the text**	**PDB No. (Chain ID)**	**PDB No. (Chain ID)**	**PDB No. (Chain ID)**	**PDB No. (Chain ID)**	**PDB No. (Chain ID)**	**PDB No. (Chain ID)**
701	603 (8)	902 (9)	703 (10)			
702	604 (8)	903 (9)	704 (10)		501 (12)	
703	605 (8)	904 (9)	705 (10)		502 (12)	501 (13)
704	606 (8)	905 (9)	706 (10)		503 (12)	
708	607 (8)	906 (9)		705 (11)	504 (12)	502 (13)
709	608 (8)	907 (9)		706 (11)	505 (12)	503 (13)
710	609 (8)	908 (9)	707 (10)	707 (11)	506 (12)	
711	610 (8)	909 (9)	708 (10)	708 (11)		
712	611 (8)	910 (9)	709 (10)	709 (11)	507 (12)	
713	612 (8)	911 (9)	710 (10)	710 (11)	508 (12)	
714	613 (8)	912 (9)	711 (10)	711 (11)		
715		913 (9)				
716	614 (8)	914 (9)	712 (10)			
**Cars in the text**						
721	615 (8)	915 (9)		712 (11)	509 (12)	
722	616 (8)	916 (9)		713 (11)	510 (12)	

Table S8 | Correspondence between the numbering of water molecules in each LHCI subunit used in the text and those in the PDB file.

	LHCI-1	LHCI-2	LHCI-3	LHCI-4	LHCI-5	LHCI-6	LHCI-7
**Water molecules in the text**	**PDB No. (Chain ID)**	**PDB No. (Chain ID)**	**PDB No. (Chain ID)**	**PDB No. (Chain ID)**	**PDB No. (Chain ID)**	**PDB No. (Chain ID)**	**PDB No. (Chain ID)**
16							801 (7)
17							802 (7)
19	601 (1)						
20	604 (1)						
21	602 (1)						
22		602 (2)					
23		601 (2)					
24		604 (2)					
25		603 (2)					
26			803 (3)				
27	603 (1)						
28			801 (3)				
29			802 (3)				
30			804 (3)				
31				803 (4)			
32				802 (4)			
33					802 (5)		
34						1001 (6)	
36					801 (5)		
43				801 (4)			

	LHCI-8	LHCI-9	LHCI-11
**Water molecules in the text**	**PDB No. (Chain ID)**	**PDB No. (Chain ID)**	**PDB No. (Chain ID)**
18	701 (8)		
35	702 (8)		
41		1001 (9)	
42		1002 (9)	
44			801 (11)

Table S9 | Correspondence between the numbering of the lipid molecule in each LHCI subunit used in the text and those in the PDB file.

	LHCI-1	LHCI-2	LHCI-3	LHCI-4	LHCI-5	LHCI-6	LHCI-7
**Lipid molecule in the text**	**PDB No. (Chain ID)**	**PDB No. (Chain ID)**	**PDB No. (Chain ID)**	**PDB No. (Chain ID)**	**PDB No. (Chain ID)**	**PDB No. (Chain ID)**	**PDB No. (Chain ID)**
731	521 (1)	521 (2)	719 (3)	714 (4)	714 (5)	917 (6)	717 (7)

	LHCI-8	LHCI-9	LHCI-10	LHCI-11
**Lipid molecule in the text**	**PDB No. (Chain ID)**	**PDB No. (Chain ID)**	**PDB No. (Chain ID)**	**PDB No. (Chain ID)**
731	617 (8)	917 (9)	713 (10)	714 (11)
